# Supplementary material for: Diverse array of neutralizing antibodies elicited upon Spike Ferritin Nanoparticle vaccination in rhesus macaques
Source: Nat Commun. 2024 Jan 3;15:200. doi: 10.1038/s41467-023-44265-0 (PMC10764318; doi:10.1038/s41467-023-44265-0)
Supplement: Supplementary file 1 — Supplementary Information [file 41467_2023_44265_MOESM1_ESM.pdf]

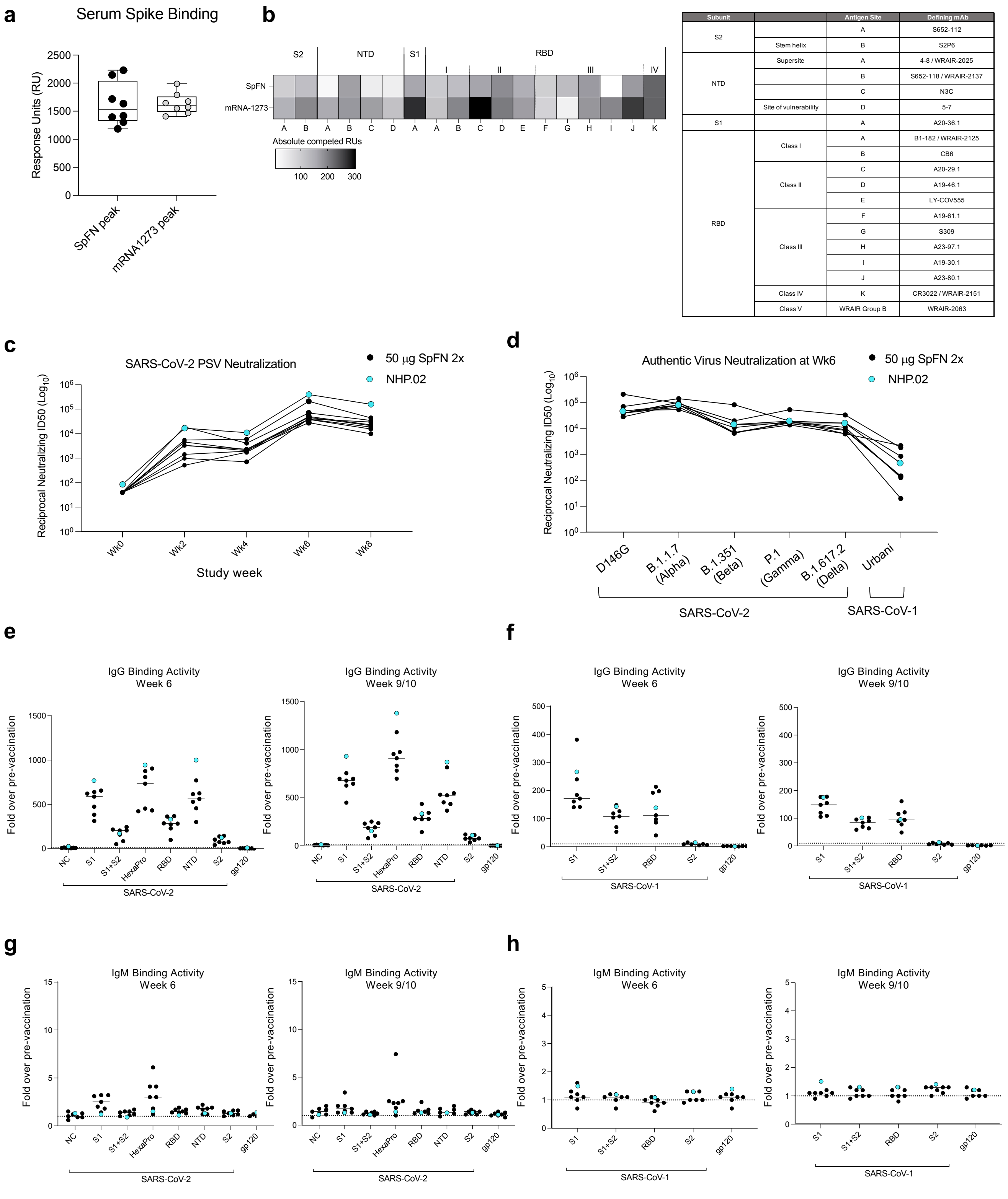


| Subunit | | Antigen Site | Defining mAb |
|---|---|---|---|
| S2 | | A | S652-112 |
| | Stem helix | B | S2P6 |
| NTD | Supersite | A | 4-8 / WRAIR-2025 |
| | | B | S652-118 / WRAIR-2137 |
| | | C | N3C |
| | Site of vulnerability | D | 5-7 |
| S1 | | A | A20-36.1 |
| RBD | Class I | A | B1-182 / WRAIR-2125 |
| | | B | CB6 |
| | Class II | C | A20-29.1 |
| | | D | A19-46.1 |
| | | E | LY-COV555 |
| | Class III | F | A19-61.1 |
| | | G | S309 |
| | | H | A23-97.1 |
| | | I | A19-30.1 |
| | | J | A23-80.1 |
| | Class IV | K | CR3022 / WRAIR-2151 |
| | Class V | WRAIR Group B | WRAIR-2063 |

**Supplementary Fig. 1 | Serum neutralizing and binding antibody activity in rhesus macaques.**

**a,** Serum antibody binding to Spike trimer in rhesus macaques (2 weeks after receiving either two doses of SpFN (n= 8 animals) or mRNA-1273 (n= 8 animals)). **b,** Competition of serum antibody binding with a panel of monoclonal antibodies to Spike subdomains to map serum antibody specificity in macaques receiving SpFN or mRNA-1273. **c**, Reciprocal serum ID50 values across study weeks 0-8 using pseudotyped SARS-CoV-2 (WA-1) neutralization assays from animals vaccinated twice with 50 µg SpFN (black). Non-human primate (NHP).02 chosen for mAb isolation is shown in aqua. **d**, Reciprocal serum ID50 values from authentic virus neutralization assays across SARS-CoV-2 VOCs and SARS-CoV-1 at the peak neutralization timepoint (week 6). Serum IgG binding antibody binding across **e**, SARS-CoV-2 and **f**, SARS-CoV-1 S protein subdomains, at study week 6 and 9/10. Serum IgM binding antibody activity across **g**, SARS-CoV-2 and **h**, SARS-CoV-1 S protein subdomains, at study week 6 and 9/10. For panels **c**-**h**, aqua denotes NHP.02.

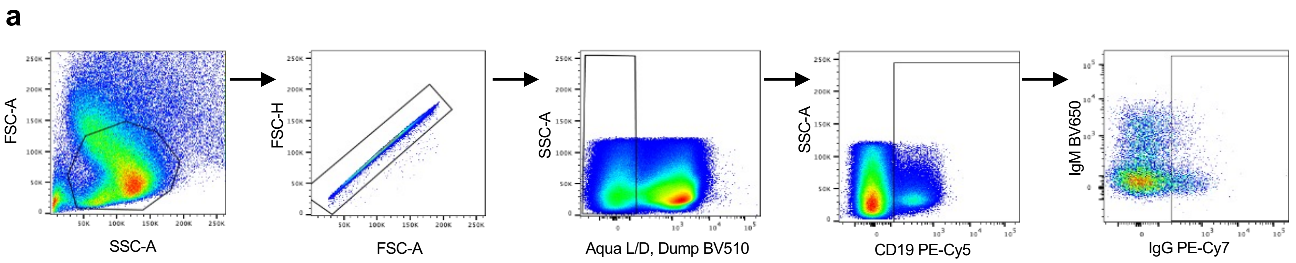


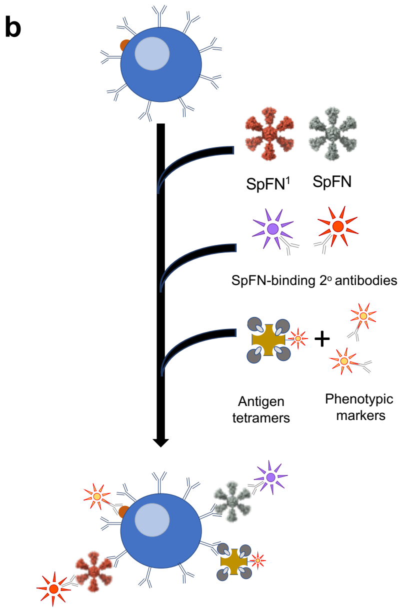


| Marker | Clone | Fluorophore | Purpose |
|---|---|---|---|
| SpFN (SARS-CoV-2) | N/A | APC | Bait |
| SpFN1 (SARS-CoV-1) | N/A | PE | Bait |
| NTD (SARS-CoV-2) | Antigenic Tetramer | BV421 | Bait |
| RBD (SARS-CoV-2) | Antigenic Tetramer | BV785 | Bait |
| CD19 | J3-119 | PE-Cy5 | B cell lineage |
| IgG | M1310G05 | PE-Cy7 | Isotype |
| IgM | MHM-88 | BV650 | Isotype |
| Live/Dead | N/A | Aqua | Viability |
| CD3 | SP34-2 | BV510 | Dump |
| CD4 | SK3 | BV510 | Dump |
| CD14 | M5E2 | BV510 | Dump |
| CD16 | 3G8 | BV510 | Dump |

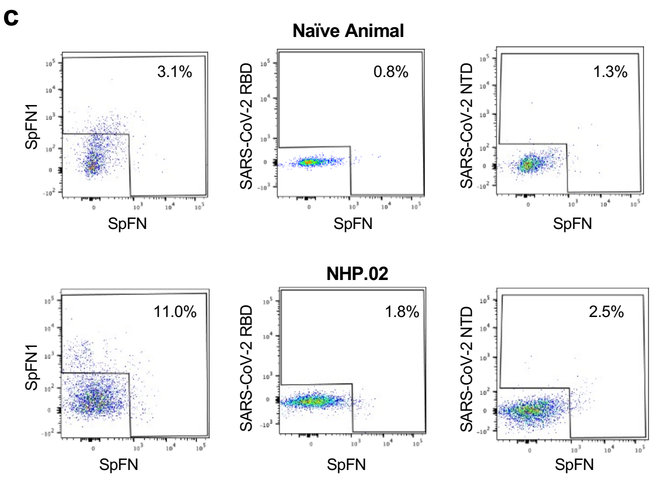


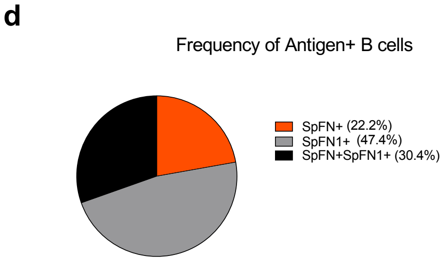


**Supplementary Fig. 2 | Sort strategy and frequency of SARS-CoV binding B cells**

**a**, Gating strategy used for the isolation of SARS-CoV-specific B cells from PBMCs at week 6, after two doses of 50 µg of SpFN. B cells were identified as lymphocytes, singlets, dump channel/viability-, CD19+, and IgG+ and IgM+IgG+. **b**, (Left) Schematic of the flow cytometry sequential staining strategy used to identify SARS-CoV-specific B cells. SpFN molecules presenting either SARS-CoV-1 spike (SpFN1) or SARS-CoV-2 spike (SpFN) were incubated with PBMCs and sequentially stained with antigenic tetramers and phenotypic markers to identify SARS-CoV specific B cells. (Right) Flow cytometry panel used to stain PBMCs from the peak neutralization time point (week 6) from NHP.02 **c**, Frequency of SARS-CoV-specific B cells identified to both SARS-CoV-1 and SARS-CoV-2 in SpFN-vaccinated macaque (NHP.02) compared to a naïve animal **d**, Quantification of the frequency of SpFN molecule reactive B cells using the SpFN and SpFN1 particles as probes during the B cell sort.

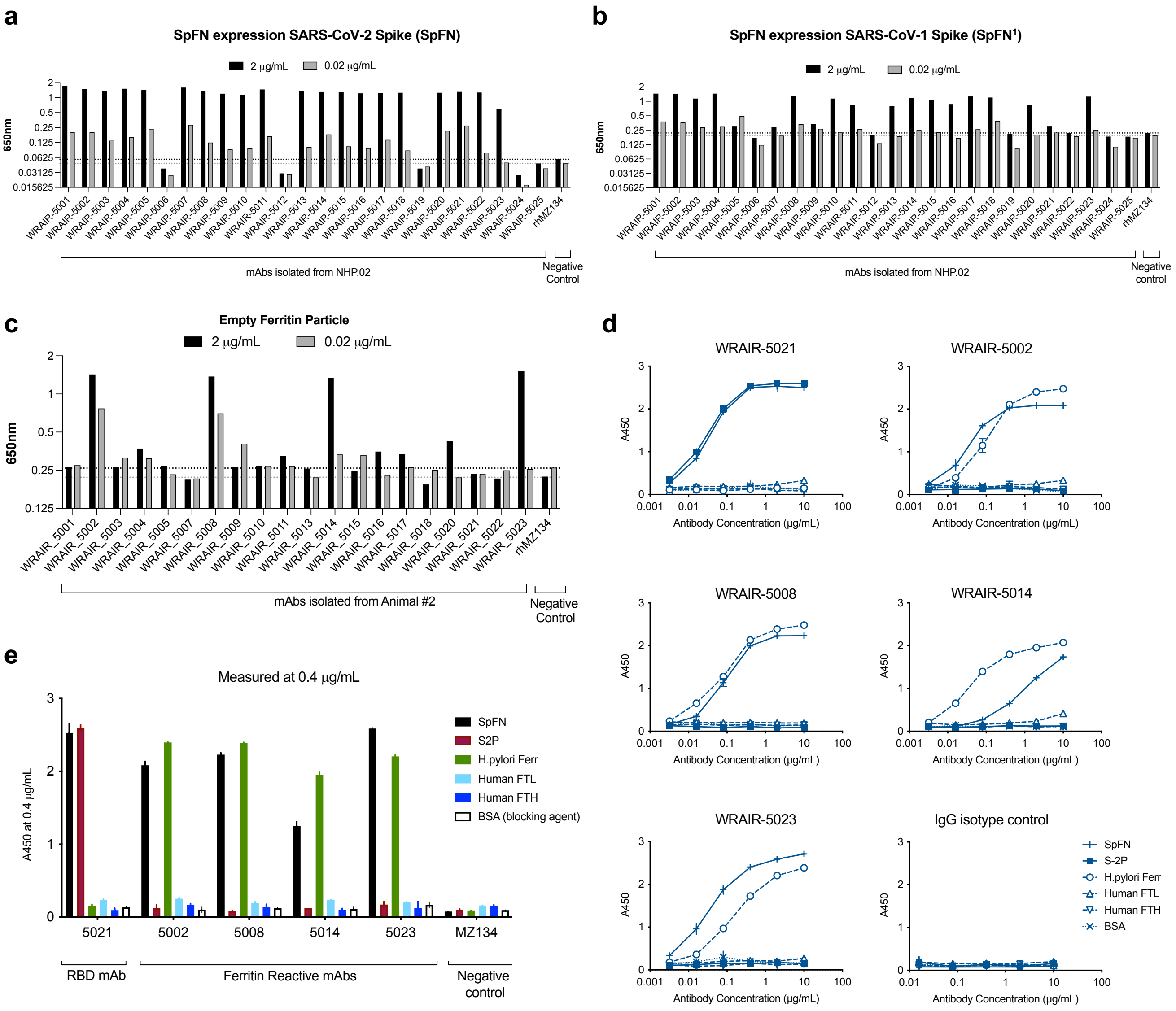


**Supplementary Fig 3 | Screening mAbs for SARS-CoV reactivity and identification of ferritin reactive mAbs**

**a-c**, Purified mAbs were tested for binding in an enzyme immunoassay (EIA) to the SpFN particle expressing **a**, SARS-CoV-2 spike (SpFN), **b**, SARS-CoV-1 spike (SpFN1), or **c**, empty ferritin particles without spike proteins. The dotted lines represent background level of binding of isotype control Zika mAb rhMZ134 when used at either 2 µg/mL (black) or 0.02 µg/mL (gray). **d**, mAbs positive in the EIA for binding to the empty ferritin particle (WRAIR-5002, WRAIR-5008, WRAIR-5014, and WRAIR-5023) were titrated for binding against the SpFN, the stabilized spike protein alone (S2P), ferritin derived from *H. pylori,* or the human ferritin light chain (FTL) or the heavy chain (FTH). BSA and the IgG isotype control (rhMZ134) were used as negative controls. WRAIR-5021 was used as the positive control mAb with known binding to SpFN and S-2P. **e**, Summary of binding of ferritin-reactive mAbs and control mAbs at a single concentration of 0.4 µg/mL.

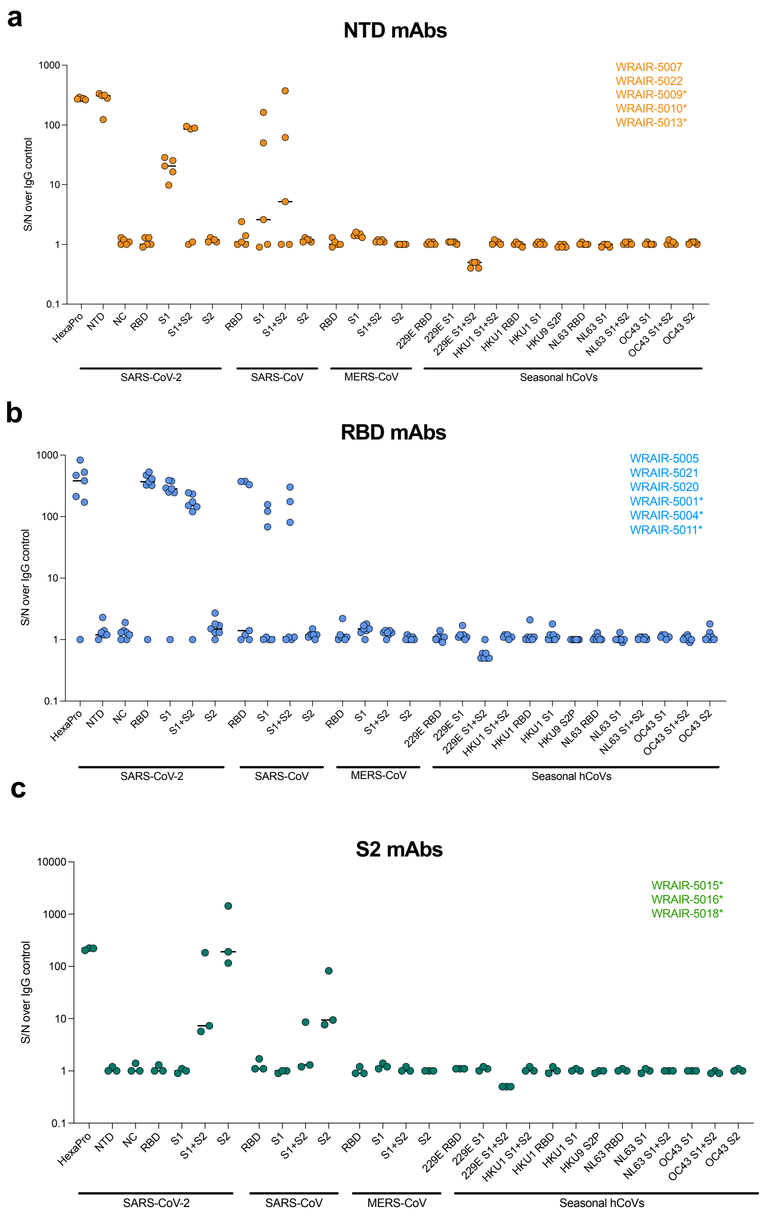


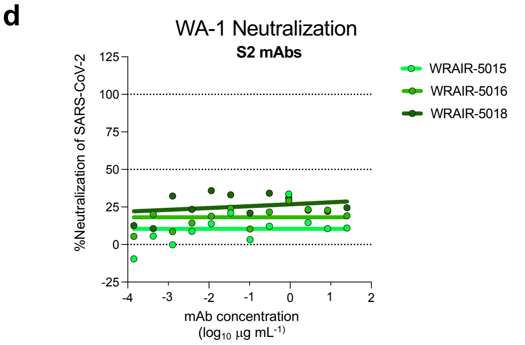


**e**

| | SARS-CoV-2 WA-1 IC50 | WA-1 IC80 | Delta (B.1.617.2) IC50 | Delta (B.1.617.2) IC80 | Omicron (BA.1) IC50 | Omicron (BA.1) IC80 | Omicron (BA.5) IC50 | Omicron (BA.5) IC80 | **SARS-CoV-1** IC50 | **SARS-CoV-1** IC80 |
|---|---|---|---|---|---|---|---|---|---|---|
| WRAIR-5009 | 0.065 | 25.000 | 25.000 | 25.000 | 25.000 | 25.000 | 25.000 | 25.000 | 25.000 | 25.000 |
| WRAIR-5010 | 0.141 | 25.000 | 25.000 | 25.000 | 25.000 | 25.000 | 25.000 | 25.000 | 25.000 | 25.000 |
| WRAIR-5013 | 0.060 | 25.000 | 25.000 | 25.000 | 25.000 | 25.000 | 25.000 | 25.000 | 25.000 | 25.000 |
| WRAIR-5018 | 25.000 | 25.000 | | | 25.000 | 25.000 | | | | |

**Supplementary Fig. 4 | Monoclonal antibody binding and neutralization**
**a-c**, Binding activity of isolated WRAIR mAbs to the S protein subdomains of SARS-CoV-2, SARS-CoV-1, MERS, and four seasonal hCoVs as measured in a multiplexed bead-based Luminex assay. Antibodies fell into distinct patterns of domain recognition, with mAbs identified as targeting the (**a**) NTD, (**b**) RBD, or (**c**) S2. WRAIR mAbs that demonstrated ability to cross-bind SARS-CoV-1 subdomains are marked with *. **d**, Neutralization curves of the S2-directed mAbs against SARS-CoV-2 WA-1. **e,** IC50 and IC80 values of NTD and S2 directed mAbs as measured through a PSV neutralization assay against SARS-CoV-2 VoCs and SARS-CoV-1.

**a**

NTD competition groups (% residual binding)

| | Antigen site | Defining mAb | Secondary mAb | | | | |
|---|---|---|---|---|---|---|---|
| | | | WRAIR-5007 | WRAIR-5022 | WRAIR-5009 | WRAIR-5010 | WRAIR-5013 |
| Primary mAb | WRAIR Group A | WRAIR-2025 | 14.3 | 8.0 | 100.0 | 95.3 | 91.8 |
| | A | 4-8 | 13.4 | 0.7 | 86.9 | 90.5 | 85.6 |
| | WRAIR Group B | WRAIR-2137 | 78.0 | 5.3 | 1.3 | 0.9 | 3.9 |
| | B | S652-118 | 46.8 | 0.4 | 0.7 | 0.9 | 0.2 |
| | C | N3C | 73.0 | 44.1 | 65.1 | 75.2 | 66.9 |
| | WRAIR Group C | WRAIR-2054 | 4.0 | 2.8 | 91.8 | 96.1 | 91.9 |
| | D | 5-7 | 75.5 | 0.0 | 13.4 | 18.1 | 22.6 |

**b**

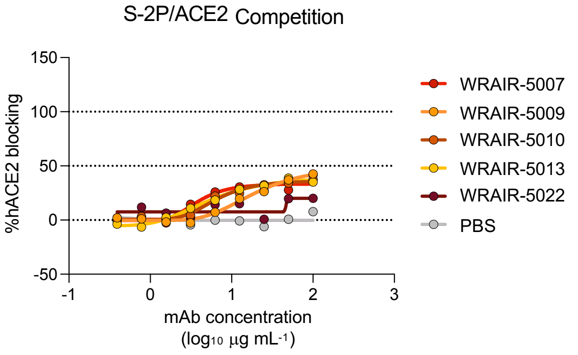


**c**

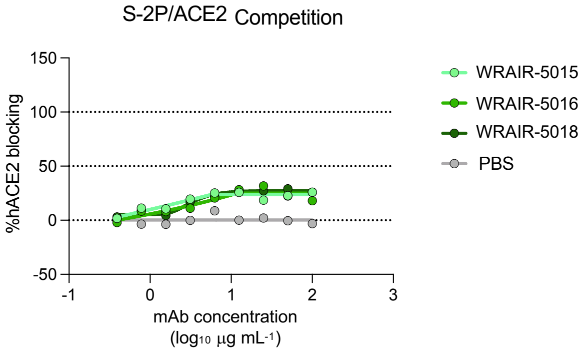


**Supplementary Fig. 5 | NTD epitope mapping and NTD- and S2-mAb ACE2 inhibition**

**a,** Epitope binning of NTD-directed mAbs as measured through a BLI-based competition assay. Values are the percentage of residual binding of the indicated WRAIR second antibody after saturation of the NTD with a representative first antibody. Competition groups based on previously defined WRAIR mAbs are indicated by boxes in shades of brown. b-c, ACE2 binding inhibition curves of isolated **(b)** NTD and **(c)** S2 mAbs for their ability to inhibit binding of the stabilized S protein (S-2P) to hACE2 as measured by BLI.

**a**

RBD competition groups (% residual binding)

| | Class | Polyclonal Antigen site | Competing mAb | Secondary mAb WRAIR-5005 | WRAIR-5021 | WRAIR-5020 | WRAIR-5001 | WRAIR-5004 | WRAIR-5011 |
|---|---|---|---|---|---|---|---|---|---|
| Primary mAb | Class I | WRAIR Group A | WRAIR-2125 | 4.9 | 0.3 | 0.2 | 46.1 | 42.5 | 47.8 |
| | | A | B1-182 | 0.1 | 2.8 | 0.9 | 48.1 | 41.5 | 49.9 |
| | | B | CB6 | 0.8 | 2.4 | 0.5 | 42.3 | 41.2 | 37.1 |
| | Class II | C | A20-29.1 | 47.0 | 48.5 | 50.9 | 47.1 | 37.9 | 40.9 |
| | | D | A19-46.1 | 35.1 | 0.7 | 0.6 | 0.0 | 0.0 | 0.3 |
| | | E | LY-COV555 | 0.5 | 0.3 | 0.0 | 50.2 | 49.2 | 55.0 |
| | Class III | F | A19-61.1 | 44.8 | 3.1 | 0.6 | 54.1 | 46.8 | 51.8 |
| | | G | S309 | 54.0 | 53.5 | 61.4 | 8.8 | 11.0 | 9.2 |
| | | H | A23-97.1 | 53.6 | 51.1 | 50.5 | 0.4 | 4.4 | 3.9 |
| | | I | A19-30.1 | 43.6 | 50.2 | 48.5 | 0.4 | 2.4 | 1.2 |
| | | J | A23-80.1 | 47.1 | 53.1 | 50.7 | 0.7 | 1.3 | 0.2 |
| | Class IV | WRAIR Group C | WRAIR-2151 | 52.2 | 56.2 | 53.2 | 47.7 | 45.4 | 44.4 |
| | | K | CR3022 | 48.3 | 46.0 | 40.9 | 40.8 | 37.7 | 36.3 |
| | Class V | WRAIR Group B | WRAIR-2063 | 51.0 | 51.0 | 3.3 | 1.1 | 3.3 | 1.1 |

**b**

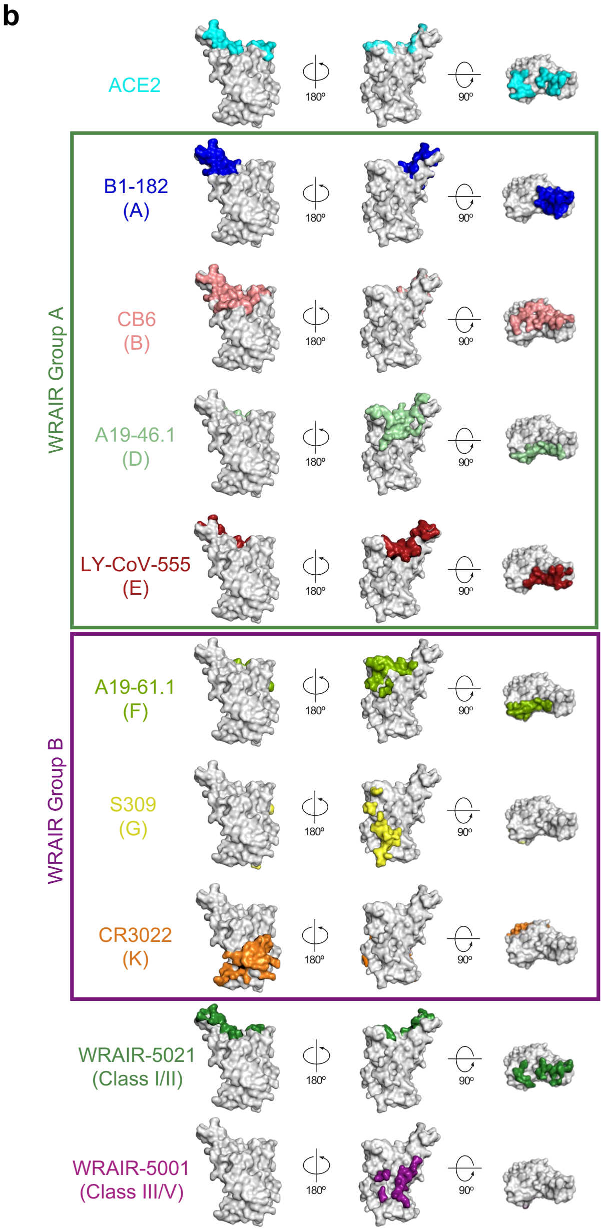


**c**

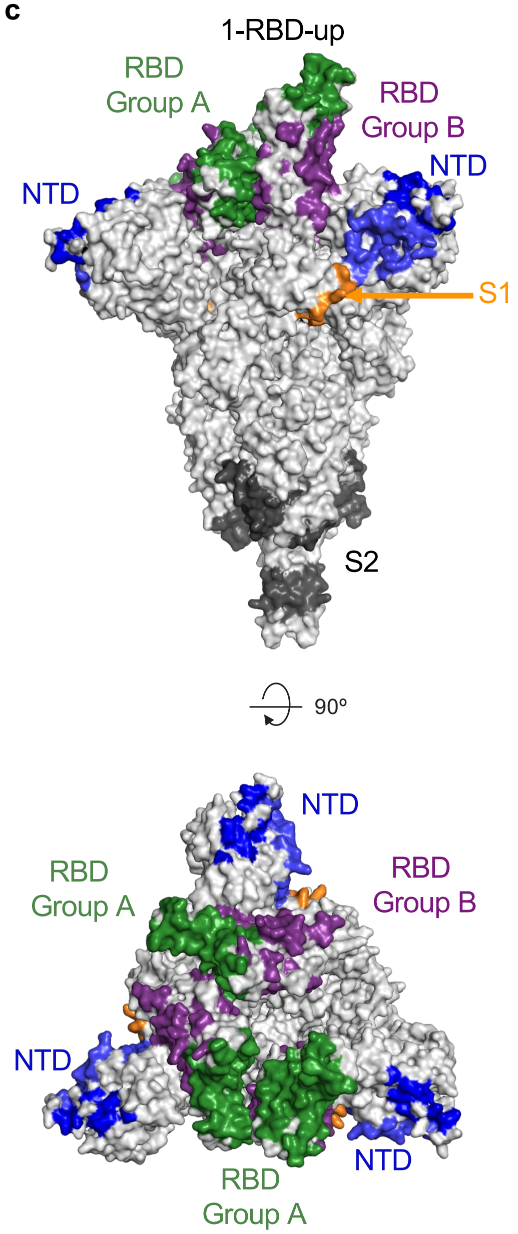


**Supplementary Fig. 6 | Antigenic surface mapping of the SARS-CoV-2 S protein and S RBD. a,** Epitope binning of RBD-directed mAbs as measured through a BLI-based competition assay. Values are the percentage of residual binding of the indicated WRAIR second antibody after saturation of the RBD with a representative first antibody. **b,** The SARS-CoV-2 RBD (gray surface representation), with epitopes outlined for the following: ACE2 (cyan, PDB 6M0J), B1-182 (blue, PDB 7MLZ), CB6 (salmon, PDB 7C01), A19-46.1 (pale green, PDB 7TC9), LY-CoV-555 (dark red, PDB 7KMG), A19-61.1 (light green, PDB 7TB8), S309 (yellow, 7R6W), CR3022 (orange, PDB 6YLA), WRAIR-5021 (green, this work), and WRAIR-5001 (purple, this work). **c,** The epitopes of antibodies used for the serum antibody competition assays, mapped onto the SARS-CoV-2 spike (PDB 7KRR): NTD (blue), S1 (orange), S2 (black), RBD group A (green), and RBD group B (purple), identifying SpFN-elicited antibody responses on the Spike trimer.

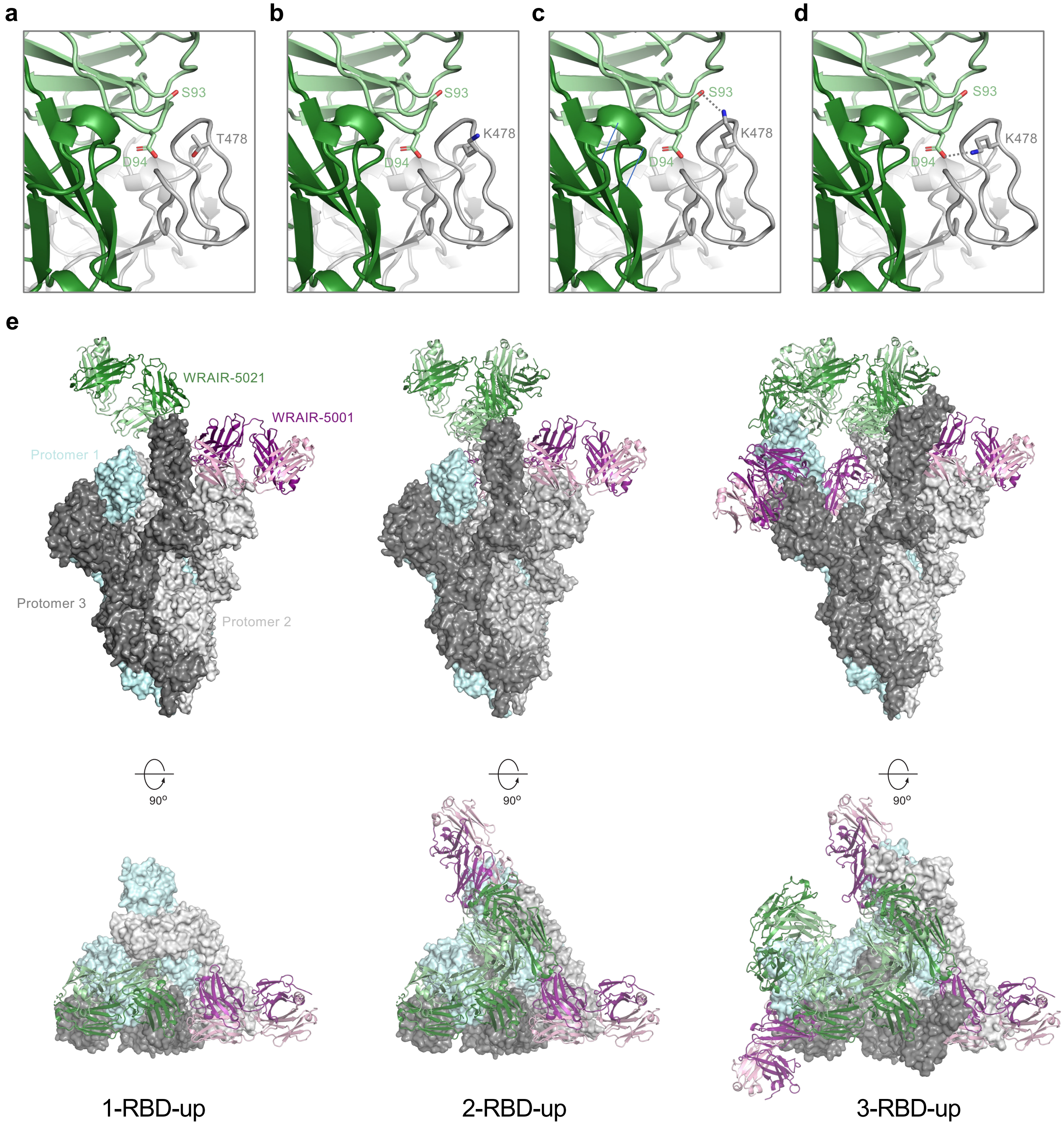


**Supplementary Fig. 7 | Proposed molecular mechanism of *in vivo* protection by WRAIR-5021 and WRAIR-5001**

**a,** The Delta variant includes the T478K mutation, which is within the WRAIR-5021 epitope. **b,** Mutation of threonine 478 to lysine would not sterically occlude RBD binding to WRAIR-5021 and could further stabilize the interface through possible salt bridge formation between the side chains of 478 and **c**, WRAIR-5021 light chain S93 or **d**, WRAIR-5021 light chain D94. **b-d** show 3 possible rotamers of the lysine butylammonium side chain. **e,** Structural superimposition of the WRAIR-5021-RBD (green) and WRAIR-5001-RBD (purple) complexes with open conformations of SARS-CoV-2 S-2P: 1-RBD-up (left; PDB: 6X2A), 2-RBD-up (center; PDB 6X2B), and 3-RBD-up (right; PDB 7T3M). Side and top views are shown.

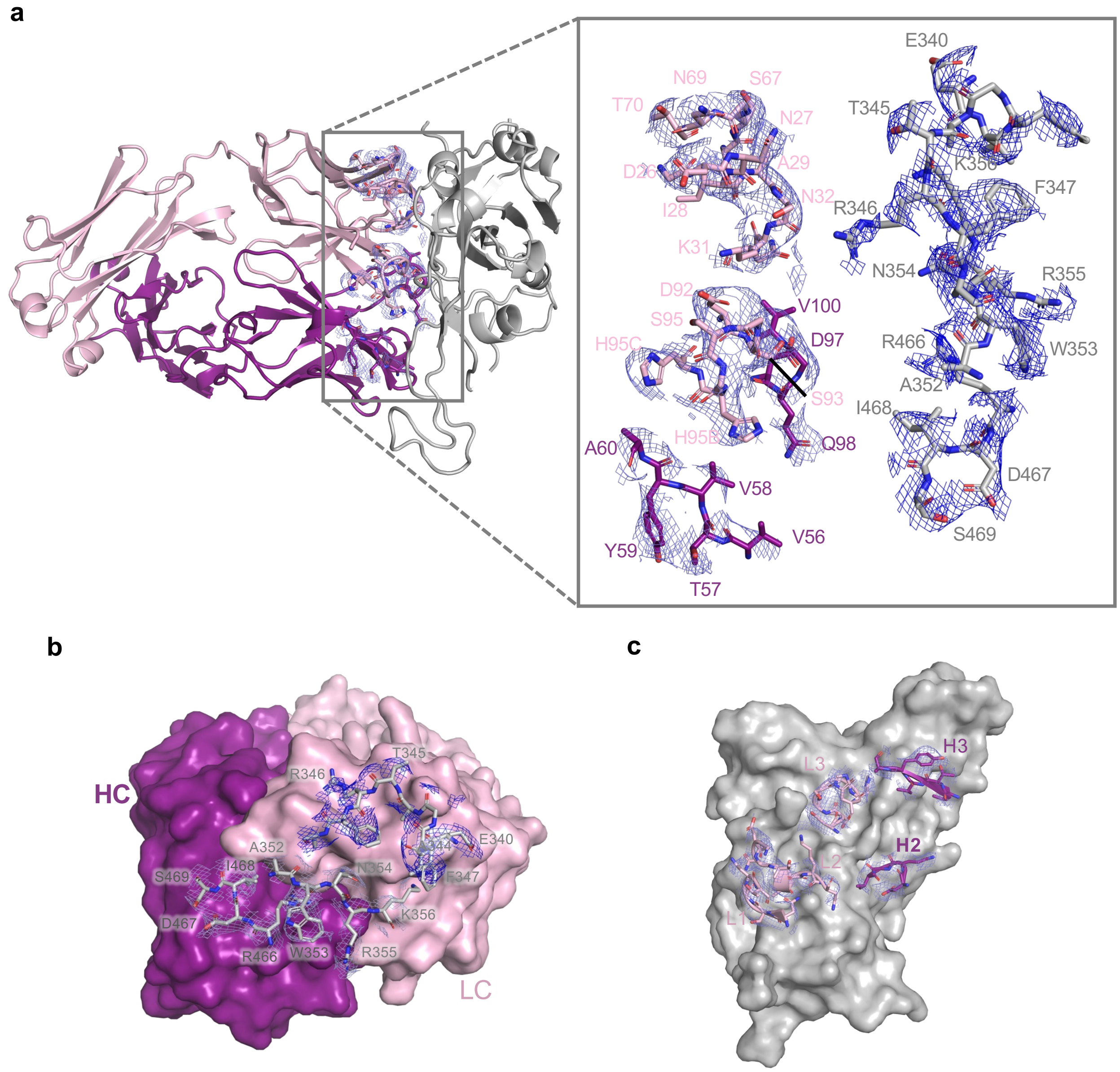


**Supplementary Fig. 8 | Electron density at the RBD-WRAIR-5001 interface.**

**a**, Representative 2Fo-Fc electron density map, contoured at 0.8 $\sigma$ (blue mesh), for residues of the RBD epitope (gray) and the WRAIR-5001 heavy (dark purple) and light (pink) chain CDRs. In the zoomed inset, the interface has been separated to better show the electron density around individual residues. **b**, The WRAIR-5001 Fab shown in surface representation, with the RBD epitope residues labeled and shown as gray sticks with associated electron density in blue mesh. **c**, The RBD shown in gray surface representation, with the WRAIR-5001 CDRs shown as both cartoon and stick representation, and the electron density surrounding these residues, in blue mesh.

**Supplementary Table 1.** Gene usage of SARS-CoV reactive mAbs isolated from SpFN vaccinated rhesus macaques.

| mAb ID | Reactivity | VH Gene | VH %SHM | CDRH3 length | VDJ Junction | VL gene | VL %SHM | CDRL3 length | VJ Junction |
|---|---|---|---|---|---|---|---|---|---|
| CoV_5001 | RBD | IGHV1-200*01 | 3.82 | 13 | CARERDQLVVYFDHW | IGLV3-36*02 or IGLV3-40*01 | 5.71 | 12 | CQVWDSYSGHHVLF |
| CoV_5002 | Ferritin | IGHV3-184*01 | 2.04 | 13 | CTRSDGSGRVYFDYW | IGLV5-83*02 | 2.61 | 8 | CMIWHNNAVF |
| CoV_5003 | Hexapro | IGHV3-136*01 | 1.74 | 15 | CTRARIRWGVVDRFDVW | IGLV1-65*01 | 5.24 | 11 | CLAWDNSLSAVLF |
| CoV_5004 | RBD | IGHV1-180*01 | 1.04 | 14 | CTRERVVAAAGYFDYW | IGLV3-34*01 | 3.57 | 11 | CQVWDSSPDHYIF |
| CoV_5005 | RBD | IGHV4-127*01 | 2.75 | 14 | CASLDYGTTYSWFDVW | IGLV3-40*01 | 1.43 | 10 | CQVWDSSSDVLF |
| CoV_5007 | NTD | IGHV4S13*01 | 3.06 | 15 | CAREDGSNVAGWYFDLW | IGLV3-40*01 | 0.71 | 10 | CQVWDSSSDHPF |
| CoV_5008 | Ferritin | IGHV4-147*01 | 3.12 | 14 | CARDPDYGYNYVLGYW | IGLV1-65*01 | 5.94 | 11 | CLAWDNSLTAHLF |
| CoV_5009 | NTD | IGHV4-65*02 | 2.41 | 20 | CARERVITIFGEVITYNWFDVW | IGLV5-69*01 | 1.95 | 9 | CAIWHSSASVF |
| CoV_5010 | NTD | IGHV4-76*01 | 5.5 | 19 | CAREIGVTIFGVVIYNWFDVW | IGLV5-83*02 | 0.98 | 9 | CMIWHNNAVLF |
| CoV_5011 | RBD | IGHV1-180*01 | 2.08 | 13 | CAGEEYSNYGYFDLW | IGLV3-34*01 | 3.21 | 11 | CQVWDSSGDHYIF |
| CoV_5013 | NTD | IGHV4-147*01 | 4.86 | 18 | CARRERITIFGVVDNWFDVW | IGLV5-62*01 | 5.86 | 9 | CAIGHNSGYIF |
| CoV_5014 | Ferritin | IGHV3-136*01 | 1.04 | 19 | CTRAEGPYYSGRYYPPDLDYW | IGLV2S7*01 | 1.38 | 10 | CCSYTTSNTFVF |
| CoV_5015 | S2 | IGHV4-127*01 | 1.72 | 14 | CARESWGHEVNSLDVW | IGLV3-2*01 | 6.07 | 13 | CAASYGSGSSWQWVF |
| CoV_5016 | S2 | IGHV4-76*01 | 6.19 | 17 | CARFGYQPDVTTYNSLDVW | IGKV1-22*01 | 3.23 | 9 | CQQYRSRPLTF |
| CoV_5017 | Hexapro | IGHV4S13*01 | 2.38 | 14 | CARDNRIAAAGTFDYW | IGKV4S1*01 | 1.68 | 9 | CQQHYSSPLSF |
| CoV_5018 | S2 | IGHV4S13*01 | 6.19 | 8 | CARAILVDVW | IGKV3-40*03 | 1.43 | 9 | CQQYNDLVLTF |
| CoV_5020 | RBD | IGHV4-147*01 | 3.12 | 13 | CASRITIFGLLRSYW | IGKV3-35*02 | 1.43 | 9 | CQQESNWPWTF |
| CoV_5021 | RBD | IGHV4-99*01 | 1.72 | 9 | CARQPPRFDVW | IGKV1-43*02 | 5.02 | 9 | CLQYDSDPFTF |
| CoV_5022 | NTD | IGHV4-127*01 | 0.69 | 21 | CATSLQYLDWLFHTDKYNRFDVW | IGKV1-66*01 | 3.23 | 9 | CQQYNDSPLTF |
| CoV_5023 | Ferritin | IGHV4S9*01 | 4.12 | 9 | CARGFNWFDVW | IGKV1-32*01 | 1.43 | 10 | CQHYNSLPMYSF |

**Supplementary Table 2.** Binding affinity constants of WRAIR RBD mAbs measured against selected RBDs, using BLI.

| | | **WA-1** | **Delta** | **Omicron BA.1** | **Omicron BA.4/5** | **SARS-CoV-1** |
|---|---|---|---|---|---|---|
| WRAIR_5001 | $K_D$ (nM) | 0.57 ± 0.012 | 0.49 ± 0.017 | 0.23 ± 0.022 | 0.92 ± 0.018 | 3.45 ± 0.028 |
| | $k_{on}$ ($M^{-1}s^{-1}$) | $2.09 \times 10^5 \pm 5.13 \times 10^2$ | $2.18 \times 10^5 \pm 7.52 \times 10^2$ | $2.05 \times 10^5 \pm 8.6 \times 10^2$ | $8.92 \times 10^4 \pm 1.87 \times 10^2$ | $1.77 \times 10^5 \pm 6.72 \times 10^2$ |
| | $k_{off}$ ($s^{-1}$) | $1.19 \times 10^{-4} \pm 2.58 \times 10^{-6}$ | $1.09 \times 10^{-4} \pm 3.64 \times 10^{-6}$ | $4.74 \times 10^{-5} \pm 4.5 \times 10^{-6}$ | $8.19 \times 10^{-5} \pm 1.6 \times 10^{-6}$ | $6.1 \times 10^{-4} \pm 4.43 \times 10^{-6}$ |
| | $\chi^2$ | 0.8048 | 1.2981 | 1.6493 | 0.2082 | 0.6673 |
| | $R^2$ | 0.9985 | 0.9964 | 0.9952 | 0.9996 | 0.9973 |
| | | **WA-1** | **Delta** | **Omicron BA.1** | **Omicron BA.4/5** | **SARS-CoV-1** |
| WRAIR_5004 | $K_D$ (nM) | 0.72 ± 0.015 | 2.44 ± 0.023 | 1.93 ± 0.027 | 3.1 ± 0.049 | 28.9 ± 0.725 |
| | $k_{on}$ ($M^{-1}s^{-1}$) | $1.99 \times 10^5 \pm 4.29 \times 10^2$ | $2.48 \times 10^5 \pm 1.11 \times 10^3$ | $2.83 \times 10^5 \pm 1.77 \times 10^3$ | $1.86 \times 10^5 \pm 1.31 \times 10^3$ | $1.58 \times 10^5 \pm 3.73 \times 10^3$ |
| | $k_{off}$ ($s^{-1}$) | $1.54 \times 10^{-4} \pm 2.26 \times 10^{-6}$ | $6.05 \times 10^{-4} \pm 4.93 \times 10^{-6}$ | $5.48 \times 10^{-4} \pm 6.82 \times 10^{-6}$ | $5.76 \times 10^{-4} \pm 8.17 \times 10^{-6}$ | $4.58 \times 10^{-3} \pm 3.88 \times 10^{-5}$ |
| | $\chi^2$ | 0.85 | 2.0303 | 4.631 | 4.4597 | 5.769 |
| | $R^2$ | 0.9976 | 0.9921 | 0.9853 | 0.9879 | 0.9103 |
| | | **WA-1** | **Delta** | **Omicron BA.1** | **Omicron BA.4/5** | **SARS-CoV-1** |
| WRAIR_5005 | $K_D$ (nM) | 1.14 ± 0.013 | 0.57 ± 0.013 | 14.9 ± 0.23 | 41.1 ± 0.74 | 154 ± 5.1 |
| | $k_{on}$ ($M^{-1}s^{-1}$) | $2.09 \times 10^5 \pm 5.51 \times 10^2$ | $2.34 \times 10^5 \pm 7.58 \times 10^2$ | $4.12 \times 10^4 \pm 3.04 \times 10^2$ | $3.82 \times 10^4 \pm 5.32 \times 10^2$ | $2.07 \times 10^4 \pm 6.5 \times 10^2$ |
| | $k_{off}$ ($s^{-1}$) | $2.38 \times 10^{-4} \pm 2.62 \times 10^{-6}$ | $1.33 \times 10^{-4} \pm 3.1 \times 10^{-6}$ | $6.15 \times 10^{-4} \pm 8.42 \times 10^{-6}$ | $1.57 \times 10^{-3} \pm 1.76 \times 10^{-5}$ | $3.18 \times 10^{-3} \pm 3.52 \times 10^{-5}$ |
| | $\chi^2$ | 1.2879 | 1.9889 | 2.7327 | 1.3565 | 0.1677 |
| | $R^2$ | 0.9981 | 0.997 | 0.9859 | 0.9489 | 0.8797 |
| | | **WA-1** | **Delta** | **Omicron BA.1** | **Omicron BA.4/5** | **SARS-CoV-1** |
| WRAIR_5011 | $K_D$ (nM) | 0.69 ± 0.013 | 0.48 ± 0.023 | 0.38 ± 0.022 | 0.42 ± 0.022 | 12.7 ± 0.11 |
| | $k_{on}$ ($M^{-1}s^{-1}$) | $2.15 \times 10^5 \pm 5.83 \times 10^2$ | $2.1 \times 10^5 \pm 9.41 \times 10^2$ | $2.23 \times 10^5 \pm 1.04 \times 10^3$ | $1.71 \times 10^5 \pm 6.33 \times 10^2$ | $1.1 \times 10^5 \pm 7.35 \times 10^2$ |
| | $k_{off}$ ($s^{-1}$) | $1.47 \times 10^{-4} \pm 2.66 \times 10^{-6}$ | $1.01 \times 10^{-4} \pm 4.83 \times 10^{-6}$ | $8.48 \times 10^{-5} \pm 4.98 \times 10^{-6}$ | $7.15 \times 10^{-5} \pm 3.78 \times 10^{-6}$ | $1.4 \times 10^{-3} \pm 7.99 \times 10^{-6}$ |
| | $\chi^2$ | 1.8942 | 2.4308 | 4.2915 | 3.3292 | 1.3235 |
| | $R^2$ | 0.9957 | 0.9925 | 0.9929 | 0.9957 | 0.9885 |
| | | **WA-1** | **Delta** | **Omicron BA.1** | **Omicron BA.4/5** | **SARS-CoV-1** |
| WRAIR_5020 | $K_D$ (nM) | 13.0 ± 0.1 | 108 ± 1.51 | 24.1 ± 0.45 | 34.5 ± 0.75 | NBD |
| | $k_{on}$ ($M^{-1}s^{-1}$) | $2.1 \times 10^5 \pm 1.51 \times 10^3$ | $5.77 \times 10^4 \pm 7.59 \times 10^2$ | $4.57 \times 10^4 \pm 5.67 \times 10^2$ | $4.78 \times 10^4 \pm 8.07 \times 10^2$ | |
| | $k_{off}$ ($s^{-1}$) | $2.73 \times 10^{-3} \pm 8.61 \times 10^{-6}$ | $6.25 \times 10^{-3} \pm 2.85 \times 10^{-5}$ | $1.1 \times 10^{-3} \pm 1.55 \times 10^{-5}$ | $1.65 \times 10^{-3} \pm 2.28 \times 10^{-5}$ | |
| | $\chi^2$ | 2.3608 | 2.5523 | 2.8493 | 0.8851 | |
| | $R^2$ | 0.9863 | 0.9586 | 0.9578 | 0.9341 | |
| | | **WA-1** | **Delta** | **Omicron BA.1** | **Omicron BA.4/5** | **SARS-CoV-1** |
| WRAIR_5021 | $K_D$ (nM) | 1.09 ± 0.007 | 3.62 ± 0.024 | 8.05 ± 0.131 | 2.63 ± 0.191 | NBD |
| | $k_{on}$ ($M^{-1}s^{-1}$) | $3.25 \times 10^5 \pm 7.08 \times 10^2$ | $3.47 \times 10^5 \pm 1.73 \times 10^3$ | $9.26 \times 10^4 \pm 8.27 \times 10^2$ | $4.75 \times 10^4 \pm 3.78 \times 10^2$ | |
| | $k_{off}$ ($s^{-1}$) | $3.53 \times 10^{-4} \pm 2.07 \times 10^{-6}$ | $1.26 \times 10^{-3} \pm 5.21 \times 10^{-6}$ | $7.45 \times 10^{-4} \pm 1.02 \times 10^{-5}$ | $1.25 \times 10^{-4} \pm 9.02 \times 10^{-6}$ | |
| | $\chi^2$ | 0.4256 | 1.9151 | 2.6359 | 0.1791 | |
| | $R^2$ | 0.996 | 0.9832 | 0.9726 | 0.9825 | |

**Supplementary Table 3.** Crystallographic data collection and refinement statistics.

| | SARS-CoV-2 WA-1 RBD + WRAIR-5001 | SARS-CoV-2 WA-1 RBD + WRAIR-5021 |
|---|---|---|
| **Data collection** | | |
| Space group | $P2_1$ | $P2_1$ |
| Cell dimensions | | |
| a,b,c (Å) | 142.5, 116.6, 142.5 | 118.9, 84.1, 119.9 |
| α,β,γ (°) | 90, 112, 90 | 90, 114, 90 |
| Resolution (Å) | 87.31 – 4.19 (7.16 - 5.69; 5.69 - 4.97; 4.97 - 4.42; 4.42 – 4.19) | 109.8 – 2.53 (2.58 – 2.53) |
| $R_{merge}$ | 0.252 (0.502; 0.833; 1.019; 2.207) | 0.119 (0.770) |
| I / σI | 1.1 (1.1; 0.7; 0.5; 0.3) | 11.4 (1.6) |
| Reflections (uni/tot) | 31815/110888 (4553/15109) | 71266/467251 (3598/15297) |
| Completeness (%) | 99.6 (99.2) | 97.8 (76.9) |
| Redundancy | 3.5 (3.3) | 6.6 (4.3) |
| CC(1/2) | 0.981 (0.824; 0.835; 0.685; 0.348) | 0.996 (0.625) |
| $R_{pim}$ | 0.158 (0.308; 0.494; 0.647; 1.427) | 0.050 (0.405) |
| **Refinement** | | |
| Resolution (Å) | 19.99 – 4.2 (4.35 – 4.2) | 66.77 – 2.53 (2.56 – 2.53) |
| Unique reflections | 30963 (3066) | 71225 (6221) |
| $R_{work}$ / $R_{free}$ * (%) | 25.4/29.8 | 20.0/25.6 |
| Ramachandran Favored/allowed/outliers | 96.1/3.9/0.0 | 96.1/3.9/0.0 |
| B-Factors ($Å^2$) | | |
| Protein | 210.7 | 54.1 |
| Ligand/ion | 229.6 | 76.9 |
| Water | 229.6 | 49.2 |
| R.m.s deviations | | |
| Bond lengths (Å) | 0.003 | 0.003 |
| Bond angles (°) | 0.63 | 0.63 |
| PDB ID | 8FI9 | 8FHY |

Values in parentheses pertain to highest-resolution shells. *$R_{free}$ was calculated using ~5% randomly selected reflections.

**Supplementary Table 4A.** WRAIR-5021 interface with SARS-CoV-2 RBD.

| | **WRAIR-5021** | **SARS2-RBD** | **Distance (Å)** |
|---|---|---|---|
| **Hydrogen bonds** | H:GLY 26[ O ] | A:GLN 498[ NE2 ] | 2.89 |
| | H:TYR 27[ OH ] | A:SER 494[ O ] | 3.22 |
| | H:TYR 27[ OH ] | A:SER 494[ OG ] | 2.82 |
| | H:SER 28[ N ] | A:TYR 449[ OH ] | 2.92 |
| | H:TYR 33[ OH ] | A:TYR 453[ OH ] | 2.43 |
| | H:TYR 33[ N ] | A:GLN 493[ OE1 ] | 2.68 |
| | H:TYR 34[ OH ] | A:GLU 484[ OE1 ] | 3.87 |
| | H:TYR 34[ OH ] | A:CYS 488[ O ] | 3.86 |
| | H:TYR 34[ OH ] | A:PHE 490[ N ] | 2.98 |
| | H:TYR 50[ OH ] | A:ASN 487[ N ] | 3.14 |
| | H:TYR 50[ OH ] | A:GLY 485[ O ] | 3.82 |
| | H:THR 54[ OG1 ] | A:GLU 484[ OE2 ] | 2.64 |
| | H:SER 56[ OG ] | A:GLU 484[ OE2 ] | 2.72 |
| | H:TYR 58[ OH ] | A:GLU 484[ O ] | 2.83 |
| | L:TYR 49[ OH ] | A:TYR 421[ OH ] | 3.88 |
| | L:ARG 53[ NH1 ] | A:ASP 420[ OD2 ] | 3.22 |
| | L:ARG 53[ NH2 ] | A:ASN 460[ OD1 ] | 2.45 |
| | L:GLU 55[ OE1 ] | A:LYS 417[ NZ ] | 3.16 |
| | L:SER 56[ OG ] | A:GLN 409[ NE2 ] | 3.41 |
| | L:TYR 91[ O ] | A:ASN 487[ ND2 ] | 2.75 |
| | L:ASP 92[ O ] | A:SER 477[ N ] | 3.60 |
| | L:ASP 92[ O ] | A:SER 477[ OG ] | 3.29 |
| **Salt bridges** | L:ARG 53[ NH1 ] | A:ASP 420[ OD2 ] | 3.22 |
| | L:GLU 55[ OE1 ] | A:LYS 417[ NZ ] | 3.16 |

**Supplementary Table 4B.** Buried surface area of WRAIR-5021 in complex with SARS-CoV-2 RBD.

| WRAIR-5021 Residue | Bond Type | Accessible Surface Area (Å²) | Buried Surface Area (Å²) |
|---|---|---|---|
| H:GLN 1 | | 196.48 | 120.82 |
| H:GLY 26 | H | 65.30 | 37.52 |
| H:TYR 27 | H | 77.43 | 64.63 |
| H:SER 28 | H | 46.71 | 14.98 |
| H:SER 31 | | 78.03 | 30.61 |
| H:GLY 32 | | 12.69 | 6.03 |
| H:TYR 33 | H | 90.50 | 77.94 |
| H:TYR 34 | H | 92.99 | 87.42 |
| H:TYR 47 | | 69.70 | 13.32 |
| H:TYR 50 | H | 51.92 | 42.83 |
| H:SER 52 | | 5.01 | 3.74 |
| H:THR 53 | | 91.29 | 11.22 |
| H:THR 54 | H | 102.71 | 29.05 |
| H:SER 56 | H | 54.36 | 20.54 |
| H:TYR 58 | H | 122.96 | 63.50 |
| H:ARG 94 | | 34.69 | 2.33 |
| H:GLN 95 | | 30.39 | 30.39 |
| H:PRO 96 | | 9.53 | 3.27 |
| H:PRO 97 | | 109.51 | 56.19 |
| H:ARG 98 | | 169.27 | 71.16 |
| H:ASP 100 | | 63.19 | 0.37 |
| L:SER 30 | | 53.50 | 0.61 |
| L:PHE 32 | | 75.81 | 40.80 |
| L:TYR 49 | H | 76.91 | 47.29 |
| L:ASP 50 | | 41.43 | 11.79 |
| L:ARG 53 | HS | 138.10 | 77.32 |
| L:LEU 54 | | 68.42 | 12.45 |
| L:GLU 55 | HS | 62.82 | 15.79 |
| L:SER 56 | H | 109.88 | 45.45 |
| L:TYR 91 | H | 63.16 | 39.72 |
| L:ASP 92 | H | 59.65 | 48.08 |
| L:SER 93 | | 42.59 | 12.90 |
| L:ASP 94 | | 136.38 | 44.76 |
| L:PHE 96 | | 138.39 | 42.62 |

H: Hydrogen Bond, HS: Salt Bridge

| SARS2 RBD Residue | Bond type | Accessible Surface Area (Å²) | Buried Surface Area (Å²) |
|---|---|---|---|
| A:GLN 409 | H | 29.91 | 16.35 |
| A:THR 415 | | 100.50 | 9.20 |
| A:GLY 416 | | 21.57 | 15.56 |

| | | | |
|---|---|---|---|
| A:LYS 417 | HS | 110.28 | 65.42 |
| A:ASP 420 | HS | 21.51 | 16.90 |
| A:TYR 421 | H | 46.72 | 39.31 |
| A:GLY 446 | | 53.48 | 0.37 |
| A:TYR 449 | H | 129.82 | 62.85 |
| A:TYR 453 | H | 37.42 | 20.16 |
| A:LEU 455 | | 48.69 | 42.55 |
| A:PHE 456 | | 62.94 | 45.09 |
| A:SER 459 | | 64.94 | 4.15 |
| A:ASN 460 | H | 93.43 | 24.05 |
| A:TYR 473 | | 45.82 | 8.82 |
| A:GLN 474 | | 78.31 | 1.10 |
| A:ALA 475 | | 44.52 | 36.33 |
| A:GLY 476 | | 30.96 | 27.82 |
| A:SER 477 | H | 106.00 | 46.96 |
| A:THR 478 | | 69.18 | 21.25 |
| A:GLU 484 | H | 102.69 | 85.78 |
| A:GLY 485 | H | 39.99 | 18.04 |
| A:PHE 486 | | 166.71 | 95.59 |
| A:ASN 487 | H | 43.34 | 31.09 |
| A:CYS 488 | H | 7.82 | 5.64 |
| A:TYR 489 | | 91.06 | 80.77 |
| A:PHE 490 | H | 117.40 | 41.36 |
| A:GLN 493 | H | 74.62 | 65.51 |
| A:SER 494 | H | 38.80 | 32.53 |
| A:TYR 495 | | 14.53 | 0.33 |
| A:GLY 496 | | 30.86 | 16.17 |
| A:GLN 498 | H | 63.84 | 34.66 |
| A:THR 500 | | 125.68 | 1.72 |
| A:ASN 501 | | 40.33 | 25.74 |
| A:GLY 502 | | 42.09 | 9.62 |
| A:TYR 505 | | 120.29 | 42.88 |

H: Hydrogen Bond, HS: Salt Bridge

**Supplementary Table 5A.** WRAIR-5001 interface with SARS-CoV-2 RBD.

| | WRAIR-5001 | SARS2-RBD | Distance (Å) |
|---|---|---|---|
| **Hydrogen bonds** | H:GLN 64[ NE2 ] | A:THR 470[ OG1 ] | 3.90 |
| | H:LEU 99[ O ] | A:ARG 466[ NH2 ] | 3.35 |
| | L:ASN 27[ ND2 ] | A:GLU 340[ O ] | 2.39 |
| | L:LYS 31[ O ] | A:LYS 356[ NZ ] | 3.02 |
| | L:ASN 32[ OD1 ] | A:ARG 357[ N ] | 2.88 |
| | L:ASN 32[ ND2 ] | A:ARG 357[ O ] | 3.49 |
| | L:ASP 51[ OD2 ] | A:LYS 356[ NZ ] | 3.44 |
| | L:SER 52[ OG ] | A:ASN 360[ ND2 ] | 3.74 |
| | L:ASP 53[ OD1 ] | A:ARG 357[ NH2 ] | 3.47 |
| | L:ASP 53[ OD2 ] | A:ARG 357[ NH2 ] | 3.57 |
| | L:GLY 68[ N ] | A:GLU 340[ OE2 ] | 3.84 |
| | L:TRP 91[ O ] | A:ASN 354[ ND2 ] | 3.62 |
| | L:SER 93[ OG ] | A:ALA 352[ O ] | 2.41 |
| | L:HIS 95B[ NE2 ] | A:TYR 351[ OH ] | 3.04 |
| **Salt bridges** | L:ASP 26[OD2 ] | A:ARG 346[ NH2 ] | 3.92 |
| | L:ASP 51[ OD2 ] | A:LYS 356[ NZ ] | 3.44 |
| | L:ASP 53[ OD1 ] | A:ARG 357[ NH2 ] | 3.47 |
| | L:ASP 53[ OD2 ] | A:ARG 357[ NH2 ] | 3.57 |

**Supplementary Table 5B.** Buried surface area of WRAIR-5001 in complex with SARS-CoV-2 RBD.

| WRAIR-5001 Residue | Bond Type | Accessible Surface Area (Å²) | Buried Surface Area (Å²) |
|---|---|---|---|
| H:TRP 50 | | 58.55 | 17.87 |
| H:GLY 55 | | 92.89 | 1.90 |
| H:VAL 56 | | 84.34 | 41.71 |
| H:THR 57 | | 43.57 | 7.53 |
| H:VAL 58 | | 55.49 | 33.35 |
| H:GLN 64 | H | 121.17 | 18.21 |
| H:ASP 97 | | 56.82 | 0.86 |
| H:GLN 98 | | 117.77 | 17.37 |
| H:LEU 99 | H | 166.91 | 125.18 |
| H:VAL 100 | | 99.54 | 39.38 |
| H:VAL 100A | | 95.28 | 13.44 |
| L:ASP 26 | S | 117.61 | 26.80 |
| L:ASN 27 | H | 95.15 | 68.01 |
| L:ALA 29 | | 40.86 | 40.71 |
| L:SER 30 | | 78.19 | 69.28 |
| L:LYS 31 | H | 20.26 | 11.73 |
| L:ASN 32 | H | 86.14 | 60.86 |
| L:TYR 50 | | 135.78 | 40.99 |
| L:ASP 51 | HS | 15.23 | 2.37 |
| L:SER 52 | H | 52.28 | 21.55 |
| L:ASP 53 | HS | 63.15 | 19.38 |
| L:ASN 66 | | 33.68 | 11.32 |
| L:SER 67 | | 79.31 | 16.25 |
| L:GLY 68 | H | 38.29 | 15.75 |
| L:TRP 91 | H | 111.48 | 61.01 |
| L:ASP 92 | | 2.94 | 2.94 |
| L:SER 93 | H | 58.14 | 56.66 |
| L:TYR 94 | | 156.09 | 129.24 |
| L:SER 95 | | 37.90 | 5.36 |
| L:GLY 95A | | 44.79 | 5.19 |
| L:HIS 95B | H | 150.23 | 90.19 |

H: Hydrogen Bond, HS: Salt Bridge

| SARS2 RBD Residue | Bond type | Accessible Surface Area (Å²) | Buried Surface Area (Å²) |
|---|---|---|---|
| A:PRO 337 | | 44.88 | 25.31 |
| A:GLU 340 | H | 119.99 | 74.92 |
| A:VAL 341 | | 24.29 | 17.99 |
| A:ASN 343 | | 118.21 | 4.89 |
| A:ALA 344 | | 35.76 | 30.29 |
| A:THR 345 | | 131.59 | 22.87 |

| | | | |
|---|---|---|---|
| A:ARG 346 | S | 203.60 | 108.82 |
| A:PHE 348 | | 26.09 | 26.09 |
| A:SER 349 | | 14.73 | 8.10 |
| A:TYR 351 | H | 28.14 | 24.08 |
| A:ALA 352 | H | 31.13 | 30.96 |
| A:TRP 353 | | 10.14 | 8.11 |
| A:ASN 354 | H | 74.98 | 68.17 |
| A:ARG 355 | | 70.86 | 56.21 |
| A:LYS 356 | HS | 102.78 | 87.51 |
| A:ARG 357 | HS | 134.15 | 89.38 |
| A:SER 359 | | 26.63 | 4.66 |
| A:ASN 360 | H | 99.87 | 23.38 |
| A:TYR 396 | | 46.82 | 9.25 |
| A:SER 399 | | 15.24 | 7.73 |
| A:ARG 457 | | 57.42 | 1.75 |
| A:PHE 464 | | 73.87 | 13.02 |
| A:GLU 465 | | 51.61 | 2.81 |
| A:ARG 466 | H | 129.56 | 59.16 |
| A:ASP 467 | | 25.78 | 1.11 |
| A:ILE 468 | | 88.46 | 46.04 |
| A:SER 469 | | 53.12 | 39.55 |
| A:THR 470 | H | 101.21 | 23.94 |
| A:GLU 471 | | 144.60 | 14.01 |
| A:LEU 492 | | 17.38 | 1.84 |

H: Hydrogen Bond, HS: Salt Bridge

**Supplementary Table 6** NHP monoclonal antibody nucleotide and amino acid sequence information.

| Raw_heavychain_nt | Raw_Heavy chain_aa | Raw Light chain_nt | Raw_Light chain_aa |
|---|---|---|---|
| **WRAIR-5001** | | | |
| CAGGTCCAGCTGGTGCAATCCGGGGCTGAGGTGAAGAAGCCT<br>GGGGCCTCAGTGAAGCTCTCCTGCAAGGCTTCCGGTTATACTT<br>TTACCAGCTACAGTATAAACTGGGTGAGACAGGCCCCTGGACA<br>AGGACTTGAGTGGATGGGATGGGTTAACCCTAGCAATGGTGT<br>TACAGTCTACGCACAGAAGTTCCAGGGCAGAGTCACCATGACC<br>AGGGACACGTCCACGAGCACAGCCTACATGGAGCTGAGCAGC<br>CTGAGATTTGAGGACACGGCCGTGTATTACTGTGCAAGAGAA<br>AGGGACCAGCTGGTCGTCTACTTTGACCACTGGGGCCAGGGA<br>GCCCTGGTCACCGTCTCCTCA | QVQLVQSGAEVKK<br>PGASVKLSCKASGY<br>TFTSYSINWVRQAP<br>GQGLEWMGWVN<br>PSNGVTVYAQKFQ<br>GRVTMTRDTSTSTA<br>YMELSSLRFEDTAV<br>YYCARERDQLVVYF<br>DHWGQGALVTVSS | TCCTATGAGCTGACTCAGCCACGTTCAGTGTCTGTGTCC<br>CCAGGACAGACGGCCAGGATCACCTGTGGGGGAGACA<br>ACATTGCAAGTAAAAATGTGCACTGGTACCAGCAGAAG<br>TTAGCGCAGGCCCCCGTGCTGGTCATCTATTATGATAGC<br>GACCGGCCCTCAGGGATCCCTGAGCGATTCTCTGGCTCC<br>AACTCAGGGAATACCGCCACCCTGACCATCAGCGGGGT<br>CGAGGCCGGGGATGAGGCTGACTACTACTGTCAGGTGT<br>GGGACAGTTATAGTGGTCATCATGTGTTATTCGGAGGA<br>GGGACCCGGCTGACCGTCCTAGGT | SYELTQPRSVSVS<br>PGQTARITCGGD<br>NIASKNVHWYQ<br>QKLAQAPVLVIYY<br>DSDRPSGIPERFS<br>GSNSGNTATLTIS<br>GVEAGDEADYYC<br>QVWDSYSGHHV<br>LFGGGTRLTVLG |
| **WRAIR-5002** | | | |
| GAGGTGCAGCTGGTGGAGTCTGGGGGAGGCTTGGTCCAGCCT<br>GGGGGGTCCCTGAGACTCTCCTGTGCAGCCTCTGGATTCACCT<br>TCAGTAACCACTACATGTACTGGGTCCGCCAGGCTCCAGGAAA<br>GGGGCTGGAGTGGGTGGGTTTCATTAGAATCAAAGCTTACGG<br>TGGGACAACAGAATACGCCGCGTCTGTGAAAGGCAGATTCAC<br>CATCTCCAGAGATGATTCCAAAAGCATCGCCTATCTGCAAATG<br>AACAGCCTGAAAACCGAGGACACGGCCGTGTATTATTGTACTA<br>GGTCTGATGGGAGTGGTAGAGTCTACTTTGACTACTGGGGCCA<br>GGGAGTCCTGGTCACCGTCTCCTCA | EVQLVESGGGLVQ<br>PGGSLRLSCAASGF<br>TFSNHYMYWVRQ<br>APGKGLEWVGFIRI<br>KAYGGTTEYAASVK<br>GRFTISRDDSKSIAY<br>LQMNSLKTEDTAV<br>YYCTRSDGSGRVYF<br>DYWGQGVLVTVSS | CAGCCTGTGCTGACTCAGCCAACCTCCCTCTCAGCATCT<br>CCTGGAGCATCAGCCAGACTCACCTGCACCTTGAGCGG<br>TGGCATCAGTGTTGGTAGTTACAGGATATTCTGGTTCCA<br>GCAGAAGCCAGGGAGTCCTCCCCGGTATCTTCTGAACT<br>ACCACATAGACTCAGATAAGTACCAGGGCTCTGGAGTC<br>CCCAGCCGCTTCTCTGGATCCAAAGATGCTTCTGCCAAT<br>GCAGGGATTTTAGTCATCTCTGGGGTCCAGTCTGAGGA<br>TGAGGCTGACTATTACTGTATGATTTGGCACAACAATGC<br>TGTGTTCGGAAGTGGCACCAAGTTGACCGTCCTCGGT | QPVLTQPTSLSAS<br>PGASARLTCTLSG<br>GISVGSYRIFWFQ<br>QKPGSPPRYLLNY<br>HIDSDKYQGSGV<br>PSRFSGSKDASA<br>NAGILVISGVQSE<br>DEADYYCMIWH<br>NNAVFGSGTKLT<br>VLG |
| **WRAIR-5003** | | | |
| GAGGTGCAGCTGGTGGAATCTGGGGGAGGCTTGGTCCAGCCT<br>GGAGGGTCCCTGAGACTCTCCTGTGCAGCCTCTGGATTCACCT<br>TCAGTAGTTACGACATGACCTGGGTCCGCCAGGCTCCGGGAAA<br>GGGGCTGGAGTGGGTCTCATATATTAGTTACACTGGTGAAACC<br>ATAAACTACGCTGACTCCGTGAAGGGCCGATTCACCATCTCCA<br>GAGACAACGCCAAGAACTCGCTGTCTCTGCAACTGAGCAGCCT<br>GAGAGCCGAGGACACGGCCGTGTATTACTGTACTAGAGCCCG<br>TATTAGGTGGGGGGTTGTGGACCGGTTCGATGTCTGGGGCCC<br>GGGAGTCCTGGTCACCGTCTCCTCA | EVQLVESGGGLVQ<br>PGGSLRLSCAASGF<br>TFSSYDMTWVRQA<br>PGKGLEWVSYISYT<br>GETINYADSVKGRF<br>TISRDNAKNSLSLQL<br>SSLRAEDTAVYYCT<br>RARIRWGVVDRFD<br>VWGPGVLVTVSS | CAGTCTGTGCTGACGCAGCCGCCCTCAGTGTCTGGGGC<br>CCCCGGGCAGAGGGTCACCATCTCGTGCACTGGGAGCA<br>GCTCCAACATTGGAGGTTATTTTGTGTCCTGGTACCAGC<br>AACTCCCAGGAACGGTCCCCAAACTCCTCATCTATGAAA<br>ATAATAAGCGACCCTCAGGGGTTTCTGACCGATTCTCTG<br>GCTCCAAGTCTGGTACCTCAGCCTCCCTAACCATCACTG<br>GACTCCAGCCTGGGGATGAGGCTGATTATTTCTGCTTAG<br>CATGGGATAACAGCCTGAGTGCTGTGTTATTCGGAGGA<br>GGGACCCGGCTGACCGTCCTAGGT | QSVLTQPPSVSG<br>APGQRVTISCTGS<br>SSNIGGYFVSWY<br>QQLPGTVPKLLIY<br>ENNKRPSGVSDR<br>FSGSKSGTSASLTI<br>TGLQPGDEADYF<br>CLAWDNSLSAVL<br>FGGGTRLTVLG |
| **WRAIR-5004** | | | |
| CAGGTGCAGCTGGTGCAGTCTGGGGCTGAGATTAAGCAGCCT<br>GGGGCCTCAGTGAAGCTCTCCTGCAAGGCTTCTGGATATACCT<br>TCACTACCTACTACATACACTGGGTGAGACAGGCCCCTGGACA<br>AGGCCTTGAGTGGATAGGACTGATCTCCCCTTACAATGGTAAC<br>AGAGGCTACGCACAGAACTTCCAGGGCAGAGTCACCATAACTA<br>CGGACACGTCCACGAGCACAGGCTACATGGAGCTGAGCAGCC<br>TGAGATCTGAGGACACAGCCGTGTATTACTGTACGAGAGAGA<br>GGGTTGTAGCAGCGGCTGGTTACTTTGACTACTGGGGCCAGG<br>GAGTCCTGGTCACCGTCTCCTCA | QVQLVQSGAEIKQP<br>GASVKLSCKASGYT<br>FTTYYIHWVRQAPG<br>QGLEWIGLISPYNG<br>NRGYAQNFQGRVT<br>ITTDTSTSTGYMELS<br>SLRSEDTAVYYCTRE<br>RVVAAAGYFDYWG<br>QGVLVTVSS | TCCTATGAGCTGACTCAGCCACCCTCGGTGTCGGTGTCC<br>CCAGGACAGACGGCCAGGATCACCTGTGGGGGAGACA<br>ACATTGGAAGTAAAAATGTGCAGTGGTACCAGCAGAAG<br>CCAGCGCAGGCCCCTGTGCTGGTCATCTATGATGATAG<br>CGAACGGCCCTCAGGGATCCCTGAGCGATTCTCTGGCT<br>CCAACTCAGGGAACACGGCCACCCTGACCATCAGCGGG<br>GTCGAGGCCGGGGATGAGGCTGGCTATTCCTGTCAGGT<br>GTGGGATAGTAGTCCTGATCATTACATCTTCGGTGCTGG<br>GACCCGGCTCACCGTCCTAGGT | SYELTQPPSVSVS<br>PGQTARITCGGD<br>NIGSKNVQWYQ<br>QKPAQAPVLVIY<br>DDSERPSGIPERF<br>SGSNSGNTATLTI<br>SGVEAGDEAGYS<br>CQVWDSSPDHYI<br>FGAGTRLTVLG |
| **WRAIR-5005** | | | |
| CAGGTGCAGCTGCAGGAGTCGGGCCCAGGACTGGTGAAGCCT<br>TCGGAGACCCTGTCCCTCACCTGCGCTGTCTCTGGTTCCTCCGT<br>CAACAGTGGTTATGGCTGGACCTGGCTCCGCCAGCCCCCAGGG<br>AAGGGGCTGGAGTGGATTGGGTATATCGGTGGTGGTAGTGGT<br>AGCACCATCTACAACCCCTCCCTCAAGAGTCGACTCACCATTTC<br>AAAAGACACGTCCAAGAACCAGTTCTCCCTGAAGCTGAGCTCT<br>GTGACCGCCGCGGACACGGCCGTGTATTACTGTGCGAGTCTAG<br>ACTACGGTACCACCTACTCCTGGTTCGATGTCTGGGGCCCGGG<br>AGTCCTGGTCACCGTCTCCTCA | QVQLQESGPGLVK<br>PSETLSLTCAVSGSS<br>VNSGYGWTWLRQ<br>PPGKGLEWIGYIGG<br>GSGSTIYNPSLKSRL<br>TISKDTSKNQFSLKL<br>SSVTAADTAVYYCA<br>SLDYGTTYSWFDV<br>WGPGVLVTVSS | TCCTATGAGCTGACTCAGCCACGCTCAGTGTCCGTGTCC<br>CCAGGACAGATGGCCAGGATCACCTGTGGGGGAGACA<br>ACATTGGAAGTAAAAGTGTGCAGTGGTACCAACACAAG<br>CCACCGCAGGCCCCTGTGCTGGTCATCTATGCTGATAGC<br>GAACGGCCCTCAGGGATCCCTGAACGATTCTCTGGCTCC<br>AACTCAGGGAACACCGCCACCCTGACCATCAGCGGGGT<br>CGAGGCCGGGGATGAGGCTGACTATTACTGTCAGGTGT<br>GGGACAGTAGTAGTGATGTCTTATTCGGAGGAGGGACC<br>CGGCTGACCGTCCTAGGT | SYELTQPRSVSVS<br>PGQMARITCGG<br>DNIGSKSVQWYQ<br>HKPPQAPVLVIYA<br>DSERPSGIPERFS<br>GSNSGNTATLTIS<br>GVEAGDEADYYC<br>QVWDSSSDVLFG<br>GGTRLTVLG |
| **WRAIR-5007** | | | |
| CAGGTGCAGCTGCAGGAGTCGGGCCCAGGAGTGGTGAAGCCT<br>TCGGAGACCCTGTCCCTCACCTGCGCTGTCTCTGGCGGCTCCAT<br>CAGCAGTGGTTACTACTTCTGGAGCTGGATCCGCCAGCCCCCA<br>GGGAAGGGGCTGGAGTGGATTGGGGGTATCTATAGTATTACT<br>GAGAATACCAACTACAGCCCCTCCCTCAAGAGTCGAGTCACCA<br>TTTCAAAAGACACGTCCAAGAACCAGTTCTCCCTAAAGCTGAG<br>CTCTGTGACCGCCACGGACACGGGCGTGTATTACTGTGCGAGA<br>GAGGACGGTAGCAATGTCGCTGGGTGGTACTTCGATCTCTGG<br>GGCCCTGGCACCCCAATCACCATCTCCTCA | QVQLQESGPGVVK<br>PSETLSLTCAVSGGS<br>ISSGYYFWSWIRQP<br>PGKGLEWIGGIYSIT<br>ENTNYSPSLKSRVTI<br>SKDTSKNQFSLKLSS<br>VTATDTGVYYCARE<br>DGSNVAGWYFDL<br>WGPGTPITISS | TCCTATGAGCTGACTCAGCCACGCTCAGTGTCCGTGTCC<br>CCAGGACAGACGGCCAGGATCACCTGTGGGGGAGACA<br>ACATTGGAAGTAAAAGTGTGCAGTGGTACCAGCAGAG<br>GCCACCGCAGGCCCCTGTGCTGGTCATCTATGCTGATAC<br>CGAACGGCCCTCAGGGATCCCTGAGCGATTCTCTGGCT<br>CCAACTCAGGGAACACCGCCACCCTGACCATCAGCGGG<br>GTCGAGGCCGGGGATGAGGCTGACTATTACTGTCAGGT<br>GTGGGACAGTAGTAGTGATCATCCATTCGGAGGAGGG<br>ACCCGGCTGACCGTCCTAGGT | SYELTQPRSVSVS<br>PGQTARITCGGD<br>NIGSKSVQWYQ<br>QRPPQAPVLVIYA<br>DTERPSGIPERFS<br>GSNSGNTATLTIS<br>GVEAGDEADYYC<br>QVWDSSSDHPF<br>GGGTRLTVLG |
| **WRAIR-5008** | | | |
| CAGGTGCAGCTGCAGGAGTCGGGCCCAGGACTGGTGAAGCCT<br>TCGGAGACCCTGTCCCTCACCTGCGCTGTCTCTGGTGGCTCCAT<br>CAGCAGTAACTACTGGAGCTGGATCCGCCAGTCCCCAGGGAA<br>GGGACTGGAGTGGATTGGCTATATCTATGGTAGTAGAGGGAG<br>CACCAGTTACAACCCCTCCCTCAAGAGTCGAGTCACCATTTCAA<br>CAGACACGTCCAAGAACCAGTTTTCCCTGAAGCTGAGCTCTGT | QVQLQESGPGLVK<br>PSETLSLTCAVSGGS<br>ISSNYWSWIRQSPG<br>KGLEWIGYIYGSRG<br>STSYNPSLKSRVTIS<br>TDTSKNQFSLKLSSV | CAGTCTGTGCTGACGCAGCCGCCCTCAGCATCTGGGGC<br>CCCCGGGCAGAGGGTCACCATCTCCTGCACTGGGAGCA<br>CCTCCAACATCGGGACGGTTTATTATGTATCCTGGTACC<br>AGCAGTTCCCAGGAACAGCCCCCAAACTCCTCATCTATG<br>AAAATAATAAGCGACCCTCAGGGGTTTCTGACCGATTCT<br>CTGGCTCCAAGTCTGGTACCTCAGCCTCCCTGACCATCA | QSVLTQPPSASG<br>APGQRVTISCTGS<br>TSNIGTVYYVSWY<br>QQFPGTAPKLLIY<br>ENNKRPSGVSDR<br>FSGSKSGTSASLTI |

| | | | |
|---|---|---|---|
| GACCGCCGCGGACACCGCCGTGTATTACTGTGCGAGAGATCCT<br>GACTACGGTTACAACTACGTTTTGGGCTACTGGGGCCAGGGA<br>GTCCTGGTCACCGTCTCCTCA | TAADTAVYYCARDP<br>DYGYNYVLGYWGQ<br>GVLVTVSS | CTGGGCTCCAGTCTGAGGATGAGGCTGATTATTACTGCT<br>TAGCATGGGATAACAGCCTGACTGCTCACTTATTCGGA<br>GGAGGGACCCGGCTGACCGTCCTAGGT | TGLQSEDEADYY<br>CLAWDNSLTAHL<br>FGGGTRLTVLG |
| **WRAIR-5009** | | | |
| CAGGTGCAGCTGCAGGAGTCGGGCCCAGGACTGGTGAAGCCT<br>TCAGAGACCCTGTCCCTCACCTGCGCTGTCTCTGGTGGCTCTAT<br>CAGCAGTAGTAACTGGTGGAGCTGGATCCGCCAGCCCCCAGG<br>GAAGGGACTGGAGTGGATTGGGCATATCAGTGGTAGTAGTGG<br>TAGCACCTACTACAACCCCTCCCTCAAGAGTCGAGTCTCCATTT<br>CAAGAGACACGTCCAAGAACCAGTTCTCCCTGAAGCTGACCTC<br>TGTGACCGCCGCGGACACGGCCGTGTATTACTGTGCGAGAGA<br>GAGGGTGATTACAATTTTTGGAGAGGTTATCACATACAACTGG<br>TTCGATGTCTGGGGCCCGGGAGTCCTGGTCACCGTCTCCTCA | QVQLQESGPGLVK<br>PSETLSLTCAVSGGS<br>ISSSNWWSWIRQP<br>PGKGLEWIGHISGS<br>SGSTYYNPSLKSRVS<br>ISRDTSKNQFSLKLT<br>SVTAADTAVYYCAR<br>ERVITIFGEVITYNW<br>FDVWGPGVLVTVS<br>S | CAGTCTGTGCTGACCCAGCCAACCTCCCTCTCAGCATCT<br>CCGGGAGCATCAGTCAGACTCAGCTGCACCTTGAGCAG<br>TGGCATCAATGTTGATAGTTACAGCATATTCTGGTACCA<br>GCAGAAGCTAGGGAGTCCTCCCCAGTACCTTCTGTACTA<br>CTACTCAGACTCAAGTAAACACCAGGGCTCTGGAGTCC<br>CCAGCCGCTTCTCTGGATCCAAAGATGCTTCAGCCAATG<br>CAGGGCTTTTACTGATCTCTGGGCTCCAGTCTGAAGATG<br>AGGCTGACTATTACTGTGCCATATGGCACAGCAGCGCTT<br>CTGTGTTCGGAAGTGGCACCAAGTTGACCGTCCTCGGT | QSVLTQPTSLSAS<br>PGASVRLSCTLSS<br>GINVDSYSIFWYQ<br>QKLGSPPQYLLYY<br>YSDSSKHQGSGV<br>PSRFSGSKDASA<br>NAGLLLISGLQSE<br>DEADYYCAIWHS<br>SASVFGSGTKLTV<br>LG |
| **WRAIR-5010** | | | |
| CAGGTGCAGCTGCAGGAGTCGGGCCCAGGACTGTTGAAGCCT<br>TCAGACACCCTGTCCCTCACCTGCGCTGTCTCTGGTGGCTCCAT<br>CAGCGGTGGTTATGGCTGGGGCTGGATCCGCCAGCCCCCAGG<br>GAAGGGGCTGGAGTGGATTGGGAGTATCTATAGTAGTAATGG<br>GAACACCTACTACAACCCCTCCCTCAAGAGTCGAGTCACCATTT<br>CAACAGACACGTCCAAGAACCAGTTCTCCCTGAAACTGAGCTC<br>TGTGACCGCCGCGGACACGGCCGTGTATTACTGTGCGAGAGA<br>GATAGGTGTTACAATTTTTGGAGTGGTTATTTATAACTGGTTCG<br>ATGTCTGGGGCCCGGGAGTCCTGGTCACCGTCTCCTCA | QVQLQESGPGLLKP<br>SDTLSLTCAVSGGSI<br>SGGYGWGWIRQP<br>PGKGLEWIGSIYSS<br>NGNTYYNPSLKSRV<br>TISTDTSKNQFSLKL<br>SSVTAADTAVYYCA<br>REIGVTIFGVVIYN<br>WFDVWGPGVLVT<br>VSS | CAGCCTGTGCTGACTCAGCCAACCTCCCTCTCAGCATCT<br>CCTGGAGCATCAGCCAGACTCACCTGCACCTTGAGCAG<br>TGGCATCAGTGTTGGTAGTTACAGGATATTCTGGTACCA<br>GCAGAAGCCAGGGAGTCCTCCCCGGTATCTTCTGAACT<br>ACCACACAGACTCAGATTACCACCAGGGCTCTGGAGTC<br>CCCAGCCGCTTCTCTGGATCCAAAGATGCTTCGGCCAAT<br>GCAGGGATTTTACTCATCTCTGGGCTCCAGTCTGAGGAT<br>GAGGCTGACTATTACTGTATGATTTGGCACAACAATGCT<br>GTGTTATTCGGAGGAGGGACCCGGCTGACCGTCCTAGG<br>T | QPVLTQPTSLSAS<br>PGASARLTCTLSS<br>GISVGSYRIFWYQ<br>QKPGSPPRYLLNY<br>HTDSDYHQGSGV<br>PSRFSGSKDASA<br>NAGILLISGLQSE<br>DEADYYCMIWH<br>NNAVLFGGGTRL<br>TVLG |
| **WRAIR-5011** | | | |
| GAGGTGCAGCTGGTGCAGTCTGGGGCTGAGATTAAGCAGCCT<br>GGGGCCTCAGTGAAGCTCTCCTGCAAGGCTTCTGGATATACCT<br>TCACTAGCTACTACATTCACTGGGTGAGACAGGCCCCTGGACA<br>AGGCCTTGAGTGGATAGGACTGATCTCCCCTTACAATGGTAAC<br>AGAGCCTACGCACAGAACTTCCAGGGCAGAGTCACCATAACTA<br>CGGACACGTCCACGAGCACAGTCTACATGGAGCTGAGCAGCC<br>TGAAATCTGAGGACACAGCCGTGTATTACTGTGCGGGAGAGG<br>AATACAGTAACTACGGGTACTTCGATCTCTGGGGCCCTGGCAC<br>CCCAATCACCATCTCCTCC | EVQLVQSGAEIKQP<br>GASVKLSCKASGYT<br>FTSYYIHWVRQAPG<br>QGLEWIGLISPYNG<br>NRAYAQNFQGRVT<br>ITTDTSTSTVYMELS<br>SLKSEDTAVYYCAG<br>EEYSNYGYFDLWG<br>PGTPITISS | TCCTATGAGCTGACTCAGCCACCCTCGGTGTCGGTGTCC<br>CCAGGACAGACGGCCAGGATCACCTGTGGGGGAGACA<br>ACATTGGAAGTAAAAATGTGCAGTGGTACCAGCAGAAG<br>CCAGCGCAGGCCCCTGTACTGGTCATCTATGATGATAGC<br>GAACGGCCCTCAGGGATCCCTGAGCGATTCTCTGGCTC<br>CAACTCAGGGAACACGGCCACCCTGACCATCAGCGGGG<br>TCGAGGCCGGGGATGAGGCTGACTATTACTGTCAGGTG<br>TGGGATAGTAGTGGTGATCATTACATCTTCGGTGCTGG<br>GACCCGGCTCACCGTCCTAGGT | SYELTQPPSVSVS<br>PGQTARITCGGD<br>NIGSKNVQWYQ<br>QKPAQAPVLVIY<br>DDSERPSGIPERF<br>SGSNSGNTATLTI<br>SGVEAGDEADYY<br>CQVWDSSGDHYI<br>FGAGTRLTVLG |
| **WRAIR-5013** | | | |
| CAGGTGCAGCTGCAGGAGTCGGGCCCAGGACTGGTGAAGCCT<br>TCAGAGACCCTGTCCCTCACTTGTGTTGTCTCTGGTGGCTCTAT<br>CAGCAGTTATTGGTGGGGCTGGATCCGCCAGCCCCCAGGGAA<br>GGGACTGGAGTGGATTGGACGTATCTCTGGTAATAGTGGGAG<br>CACCAGCTACAACCCCTCCCTCAAGAGTCGAGTCACCATTTCAA<br>CAGACACGTCCAAGAACCAGTTCTCCCTGAAGGTGAGCTCTGT<br>GACCGCCGCGGACACGGCCGTGTATTACTGTGCGAGAGAGA<br>ACGTATTACAATTTTTGGAGTGGTCGATAACTGGTTCGATGTCT<br>GGGGCCCGGGAGTCCTGGTCACCGTCTCCTCA | QVQLQESGPGLVK<br>PSETLSLTCVVSGGS<br>ISSYWWGWIRQPP<br>GKGLEWIGRISGNS<br>GSTSYNPSLKSRVTI<br>STDTSKNQFSLKVSS<br>VTAADTAVYYCARR<br>ERITIFGVVDNWFD<br>VWGPGVLVTVSS | CAGCCTGTGCTGACTCAGCCGGCCTCCCTCTCAGCATCT<br>CCTGGAGCATCAGCCAGTCTCACATGCACCTTCAGCGGT<br>GGCATCAATGTTGCTGACTACTACATACACTGGTACCAG<br>CAGAAGCCAGGGAGTCCTCCCCGGTACCTTCTGAGGTA<br>CAAATCAGGCTCAGATTACCACCAGGGCTCTGGAGTCC<br>CCAGCCGCTTCTCTGGATCCAGAGATGCTTCGGCCAACA<br>CAGGGATTTTACGCATCTCTGGGCTCCAGTCTGAGGAT<br>GAGACTGACTATTACTGTGCCATTGGACACAACAGCGG<br>TTACATCTTCGGTGCTGGGACCCGGCTCACCGTCCTCGG<br>T | QPVLTQPASLSAS<br>PGASASLTCTFSG<br>GINVADYYIHWY<br>QQKPGSPPRYLL<br>RYKSGSDYHQGS<br>GVPSRFSGSRDA<br>SANTGILRISGLQS<br>EDETDYYCAIGH<br>NSGYIFGAGTRLT<br>VLG |
| **WRAIR-5014** | | | |
| GAGGTGCAGCTGGTGGAGTCTGGGGGAGGCTTGGTCCAGCCT<br>GGAGGGTCCCTGAGACTCTCCTGTGCAGCCTCTGGATTCACCT<br>TCAGAAGTTACGACATGAGCTGGGTCCGCCAGGCTCCGGGAA<br>AGGGGCTGGAGTGGGTCTCATATACTAGTTACACTGGTAAAAC<br>CATATACTACGCTGACTCCGTGAAGGGCCGATTCACCATCTCCA<br>GAGACAACGCCAAGAACTCGCTGTCTCTGCTAATGAGCAGCCT<br>GAGAGCCGAGGACACGGCCGTGTATTACTGTACTAGAGCCGA<br>AGGTCCCTACTATAGTGGTCGTTATTACCCCCCAGATCTTGACT<br>ACTGGGGCCAGGGAGTCCTGGTCACCGTCTCCTCA | EVQLVESGGGLVQ<br>PGGSLRLSCAASGF<br>TFRSYDMSWVRQA<br>PGKGLEWVSYTSYT<br>GKTIYYADSVKGRF<br>TISRDNAKNSLSLL<br>MSSLRAEDTAVYYC<br>TRAEGPYYSGRYYP<br>PDLDYWGQGVLVT<br>VSS | CAGTCTGTCCCGACTCAGCCTCCCTCTGTGTCTGGGTCT<br>CCTGGACAGTCGGTCACCATCTCCTGCACTGGAACCAGC<br>AGTGACATTGGTGGTTATACCTATGTCTCCTGGTACCAG<br>CAACACCCAGGCAAAGCCCCCAAACTCATGATTTATGGT<br>GTCAGTAATCGGCCCTCAGGGGTCTCTGATCGCTTCTCT<br>GGCTCCAAGTCTGGCAACACGGCCTCCCTGACCATCTCT<br>GGGCTCCAGGCTGAGGACGAGGCTGATTATTACTGTTG<br>TTCATATACAACCAGTAACACTTTTGTGTTCGGAGTTGG<br>CACCAAGTTGACCGTCCTCGGT | QSVPTQPPSVSG<br>SPGQSVTISCTGT<br>SSDIGGYTYVSWY<br>QQHPGKAPKLMI<br>YGVSNRPSGVSD<br>RFSGSKSGNTASL<br>TISGLQAEDEADY<br>YCCSYTTSNTFVF<br>GVGTKLTVLG |
| **WRAIR-5015** | | | |
| CAGGTGCAGCTGCAGGAGTCGGGCCCAGGACTGGTGAAGCCT<br>TCGGAGACCCTGTCCCTCACCTGCGCTGTCTCTGGTTACTCCAT<br>CAGCAGTGGTTATGGCTGGAGCTGGATCCGCCAGCCCCCAGG<br>GAAGGGGCTGGATTGGATTGGGTATATCGGTGGTAGTGGTGA<br>TAGCACCAACTACAACCCCTCCCTCAAGAGTCGAGTCACCATTT<br>CCAAAGACACGTCCAAGAACCAGTTCTCCCTGAAGCTGCGCTC<br>TGTGACCGCCGCGGACACGGCCGTGTATTACTGTGCGAGAGA<br>ATCTTGGGGTCACGAAGTGAACTCATTGGATGTCTGGGGCCG<br>GGGAGTTCTGGTCACCGTCTCCTCA | QVQLQESGPGLVK<br>PSETLSLTCAVSGYS<br>ISSGYGWSWIRQPP<br>GKGLDWIGYIGGSG<br>DSTNYNPSLKSRVTI<br>SKDTSKNQFSLKLRS<br>VTAADTAVYYCARE<br>SWGHEVNSLDVW<br>GRGVLVTVSS | TCCTTTGAGCTGACTCAGCCACCCTCAGTGTCGGTGTCC<br>CCAGGACAGACAGCCAGCATCACCTGCTCTGGAAATCA<br>ATTGGGGAATATATATGCTTACTGGTACCAGCAGAAGC<br>CAGGCCAGGCCCCTATACTGGTCATCTACAAAGATAGC<br>AACCGGCCCTCAGGGATCCCTGAGCGATTCTCTGGCTCC<br>AACTCAGGGAACACGGCCACCCTGACCATCAGTAGGGT<br>CGAGGCTGGGGATGAGGCCGACTATTACTGTGCTGCAA<br>GCTATGGCAGTGGGAGCAGCTGGCAGTGGGTATTCGG<br>CGGAGGGACCCGGCTGACCGTCCTAGGT | SFELTQPPSVSVS<br>PGQTASITCSGN<br>QLGNIYAYWYQQ<br>KPGQAPILVIYKD<br>SNRPSGIPERFSG<br>SNSGNTATLTISR<br>VEAGDEADYYCA<br>ASYGSGSSWQW<br>VFGGGTRLTVLG |
| **WRAIR-5016** | | | |
| CAGGTGCAGCTGCAGGAGTCGGGCCCAGGACTGTTGAAGCCT<br>TCAGACACCCTGTCCCTCACCTGCGCTGTCTCTGGTGGCTCCAT<br>CAGCGGTGGTTATGGCTGGGGCTGGATCCGCCAGCCCCCAGG<br>GAAGGGACTGGAGTGGATTGGGAGTATCTATAGTAGTGATGG<br>GAACACCTACTACAACCCCTCCCTCAAGAGTCGAGTCACCATTT<br>CAACAGACACGTCCAAGAACCAGTTCTCCCTGAAACTGAGCTC | QVQLQESGPGLLKP<br>SDTLSLTCAVSGGSI<br>SGGYGWGWIRQP<br>PGKGLEWIGSIYSS<br>DGNTYYNPSLKSRV<br>TISTDTSKNQFSLKL | GACATTCAGATGACCCAGTCTCCATCCTCCCTGTCTGCA<br>TCTGTAGGAGACACAGTCACCATCACTTGCCGGGCGAG<br>TCAGAGTATTAGCTGGTGGTTAGCCTGGTATCAGCAGA<br>AACCAGGGAAAGCCCCTAAGCCCCTGATCTATAAGGCG<br>TCCAGTTTGCAAAGTGGGGTCCCATCAAGGTTCAGCGG<br>CAGTGGATCTGGGACAGATTTCACTCTCACCATCAGCAG | DIQMTQSPSSLSA<br>SVGDTVTITCRAS<br>QSISWWLAWYQ<br>QKPGKAPKPLIYK<br>ASSLQSGVPSRFS<br>GSGSGTDFTLTIS |

| | | | |
|---|---|---|---|
| TGTGACCGCCGCGGACACGGCCGTGTATTACTGTGCGAGATTT<br>GGGTATCAACCCGACGTAACCACATACAACTCATTGGATGTCT<br>GGGGCCGGGGAGTTCTGGTCACCGTCTCCTCA | SSVTAADTAVYYCA<br>RFGYQPDVTTYNSL<br>DVWGRGVLVTVSS | CCTGCAGTCTGAAGATTTTGCAACTTATTACTGTCAACA<br>GTATAGGAGTAGACCCCTCACTTTCGGCGGAGGGACCA<br>AGGTGGAGATCAAACGG | SLQSEDFATYYCQ<br>QYRSRPLTFGGG<br>TKVEIKR |
| **WRAIR-5017** | | | |
| CAGGTGCAGCTGCAGGAGTCGGGCCCAGGAGTGGTGAAGCCT<br>TCGGAGACCCTGTCCCTCACCTGCGCTGTCTCTGGCGGCTCCAT<br>CAGCGGTGGTTACTACTACTGGAGCTGGATCCGCCAGCCCCCA<br>GGGAAGGGGCTGGAGTGGATTGGGAGTATCTATAGTAATAGT<br>GAGACTACCAACTACAGCCCCTCCCTCAAGAGTCGAGTCACCA<br>TTTCAAAAGACACGTCCAAGAACCAGTTCTCCCTAAAGCTGAG<br>CTCTGTGACCGCCACGGACACGGCCGTGTATTACTGTGCGAGG<br>GATAACCGTATAGCGGCAGCTGGAACGTTCGACTACTGGGGC<br>CAGGGAGTCCTGGTCACCGTCTCCTCA | QVQLQESGPGVVK<br>PSETLSLTCAVSGGS<br>ISGGYYYWSWIRQP<br>PGKGLEWIGSIYSN<br>SETTNYSPSLKSRVT<br>ISKDTSKNQFSLKLS<br>SVTATDTAVYYCAR<br>DNRIAAAGTFDYW<br>GQGVLVTVSS | GATATTGTGATGACCCAGTCTCCAGACTCCCTGGCTGTG<br>TCTCTGGGAGAGAGGGTCACCATCAACTGCAAGTCCAG<br>CCAGAGTCTTTTATACAGCTCCAACAATAAGAACTACTT<br>AGCCTGGTACCAGCAGAAACCAGGACAGGCTCCTAAGC<br>TGCTCATTTACTGGGCATCTACCCGGGAATCCGGGGTCC<br>CTAACCGATTCAGTGGCAGCGGGTCTGGGACAGATTTC<br>ACTCTCACCATCAGTGGCCTGCAGGCTGAAGATGTGGC<br>AGTTTATTACTGTCAGCAGCATTACAGCTCTCCTCTCAGT<br>TTTGGCCAGGGGACCAAAGTGGAGATCAAACGG | DIVMTQSPDSLA<br>VSLGERVTINCKS<br>SQSLLYSSNNKNY<br>LAWYQQKPGQA<br>PKLLIYWASTRES<br>GVPNRFSGSGSG<br>TDFTLTISGLQAE<br>DVAVYYCQQHYS<br>SPLSFGQGTKVEI<br>KR |
| **WRAIR-5018** | | | |
| CAGGTGCAGCTGCAGGAGTCGGGCCCAGGACTGGTGAAGCCT<br>TCGGAGACCCTGTCCCTCACTTGCGCTGTCTCTGGTGGCTCCAT<br>CAGCAGTAGTAACTGGTGGAGCTGGATCCGCCAGCCCCCAGG<br>GAAAGGACTGGAGTGGATTGGGGGTATCTATAGTGATACTGA<br>GAGTACCAATTACAACCCCTCCCTCGAGAGTCGAGTCACCATTT<br>CAAAAGACACGTCCAAGAACCAGTTCTCCTTGAAGCTGAGCTC<br>TGTGACCGCCGCGGACACGGCCATATATTACTGTGCGAGGGCC<br>ATTCTGGTGGATGTCTGGGGCCGGGGAGTTCTGGTCACCGTCT<br>CCTCA | QVQLQESGPGLVK<br>PSETLSLTCAVSGGS<br>ISSSNWWSWIRQP<br>PGKGLEWIGGIYSD<br>TESTNYNPSLESRVT<br>ISKDTSKNQFSLKLS<br>SVTAADTAIYYCAR<br>AILVDVWGRGVLV<br>TVSS | GAAATTGTAATGACGCAGTCTCCAGCCACCCTGTCTTTG<br>TCTCCAGGGGAAACAGCCACCCTCTCCTGCAGGGCCAG<br>TCAGAGTGTTGGCAGCTACTTAGCCTGGTACCAGCAGA<br>AACCTGGGCAGTCTCCCAAGCTCCTCGTCCATAGTGCAT<br>CCTTCAGGGCCACTGGCATCCCAGACAGGTTCAGTGGC<br>AGCGGGTCTAGGACAGAGTTCACTCTCACCATTAGCAG<br>CCTGGAGCCTGAGGATGTTGGAGTTTATCACTGTCAGC<br>AGTATAATGACTTGGTTCTCACTTTCGGCGGAGGGACC<br>AAGGTGGAGCTCAAACGA | EIVMTQSPATLSL<br>SPGETATLSCRAS<br>QSVGSYLAWYQ<br>QKPGQSPKLLVH<br>SASFRATGIPDRF<br>SGSGSRTEFTLTIS<br>SLEPEDVGVYHC<br>QQYNDLVLTFGG<br>GTKVELKR |
| **WRAIR-5020** | | | |
| CAGGTGCAGCTGCAGGAGTCGGGCCCAGGACTGGTGAAGCCT<br>TCAGAGACCCTGTCCCTCACCTGCGCTGTCTCTGGTGCCTCTAT<br>CAGCAGTTACTGGTGGGGCTGGATCCGCCAGCCCCCAGGGAA<br>GGGACTGGAGTGGATTGGACGTATCTCTGGTAGTAGTGGGAG<br>CACCAGCTACAACCCCTCCCTCAAGAGTCGAGTCACCATTTCAA<br>CAGACACGTCCAAGAACCAGTTCTCCCTGAAGCTGAGCTCTGT<br>GACCGCCGCGGACACGGCCGTGTATTACTGTGCCTCACGTATT<br>ACAATTTTTGGACTGCTCAGGAGCTACTGGGGCCAGGGAGTCC<br>TGGTCACCGTCTCCTCA | QVQLQESGPGLVK<br>PSETLSLTCAVSGAS<br>ISSYWWGWIRQPP<br>GKGLEWIGRISGSS<br>GSTSYNPSLKSRVTI<br>STDTSKNQFSLKLSS<br>VTAADTAVYYCASR<br>ITIFGLLRSYWGQG<br>VLVTVSS | GAAATTGTGATGACGCAGTCTCCAGCCACCCTGTCTTTG<br>TCTCCAAGAGAAAGAGCCACCCTCTCCTGTAGGGCCAG<br>TCAGAGTGTCAGCAGCAACTTAGCCTGGTACCAGCAGA<br>AACCTGGGCAAGCTCCCAGGCTCCTCATCTATTATGCAT<br>CCAACAGGGCCACTGGTATCCCAGACAGGTTCAGTGGC<br>AGTGGGTCTGGGACAGACTTCACTCTCACCATCAGCAG<br>CCTGGAGCCTGAAGATGTTGGACTTTATTACTGTCAGCA<br>GGAGAGTAACTGGCCGTGGACGTTCGGCCAAGGGACC<br>AAGGTGGAAATCAAACGA | EIVMTQSPATLSL<br>SPRERATLSCRAS<br>QSVSSNLAWYQ<br>QKPGQAPRLLIYY<br>ASNRATGIPDRFS<br>GSGSGTDFTLTIS<br>SLEPEDVGLYYCQ<br>QESNWPWTFGQ<br>GTKVEIKR |
| **WRAIR-5021** | | | |
| CAGGTGCAGCTGCAGGAGTCGGGCCCAGGACTGGTGAAGCCT<br>TCGGAGACCCTGTCCCTCACCTGCGCTGTCTCTGGTTACTCCAT<br>CAGCAGTGGTTACTACTGGGGCTGGATCCGTCAGCCCCCAGG<br>GAAGGGACTGGAGTATATTGGGTATTTCAGTGGTACTACTGG<br>GAGCACCTACTACAACCCCTCCCTCAAGAGTCGAGTCACCATTT<br>CAAAAGACACGTCCAAGAACCAGTTCTCCCTGAAGCTGAACTC<br>TGTGACCGCCGCGGACACGGCGGTGTATTACTGTGCGAGACA<br>GCCCCCCCGGTTCGATGTCTGGGGCCCGGGAGTCCTGGTCACC<br>GTCTCCACA | QVQLQESGPGLVK<br>PSETLSLTCAVSGYS<br>ISSGYYWGWIRQPP<br>GKGLEYIGYFSGTT<br>GSTYYNPSLKSRVTI<br>SKDTSKNQFSLKLN<br>SVTAADTAVYYCAR<br>QPPRFDVWGPGVL<br>VTVST | GACATTCAGATGACCCAGTCTCCATCTTCCCTGTCTGCAT<br>CTGTGGGAGACACAGTCACCATCACTTGCAGGGCAAGT<br>CAGGGCATTAGCAATTTTTTAAATTGGTATCAGCAGAAA<br>CCAGGAAAAGCTCCTAAGCTTCTGATCTATGATGCGTCC<br>AGATTGGAAAGTGGGGTCCCATCAAGGTTCAGTGGCAG<br>TGGATCTGGGACAGAATTCACTCTCACCATCAGCAGCCT<br>GCAGCCAGAAGATTTTGCAACTTATCACTGTCTACAGTA<br>TGACAGTGACCCTTTCACTTTCGGCCCCGGGACCAAACT<br>GGATATCAAACGA | DIQMTQSPSSLSA<br>SVGDTVTITCRAS<br>QGISNFLNWYQQ<br>KPGKAPKLLIYDA<br>SRLESGVPSRFSG<br>SGSGTEFTLTISSL<br>QPEDFATYHCLQ<br>YDSDPFTFGPGTK<br>LDIKR |
| **WRAIR-5022** | | | |
| CAGGTGCAGCTGCAGGAGTCGGGCCCAGGACTGGTGAAGCCT<br>TCGGAGACCCTGTCCCTCACCTGCGCTGTCTCTGGTTACTCCAT<br>CAGCAGTGGTTATGGCTGGAGCTGGATCCGCCAGCCCCCAGG<br>GAAGGGGCTGGAGTGGATTGGGTATATCGGTGGTAGTAGTGG<br>TAGCACCAACTACAACCCCTCCCTCAAGAGTCGAGTCACCATTT<br>CAAAGGACACGTCCAAGAACCAGTTCTCCCTGAAGCTGAGCTC<br>TGTGACCGCCGCGGACACGGCCGTATATTACTGTGCGACCTCC<br>CTTCAATATTTGGACTGGTTATTCCATACGGATAAATACAACCG<br>GTTCGATGTCTGGGGCCCGGGAGTCCTGGTCACCGTCTCCTCA | QVQLQESGPGLVK<br>PSETLSLTCAVSGYS<br>ISSGYGWSWIRQPP<br>GKGLEWIGYIGGSS<br>GSTNYNPSLKSRVTI<br>SKDTSKNQFSLKLSS<br>VTAADTAVYYCATS<br>LQYLDWLFHTDKY<br>NRFDVWGPGVLVT<br>VSS | GACATTCAGATGACGCAGTCTCCATCCTCCCTGTCTGCA<br>TCTGTAGGAGACAGAGTCACCATCACTTGCCGGGCGAG<br>TCAGGGCATTAACAATTATTTAAGTTGGTATCAGCAGAA<br>ATCAGGGAAAGCCCCTAAGCCCCTGATCTATTCTGCATC<br>CAGTTTGGAAACAGGGGTACCATCAAGGTTCAGTGGAA<br>GTAGATCTGGGACAGATTATACTCTCACCATCACCAGTC<br>TGCAGCCTGAAGATATTGCAACATATTACTGTCAACAGT<br>ATAATGATTCCCCGCTCACTTTCGGCGGAGGGACCAAG<br>GTGGAGATCAAACGG | DIQMTQSPSSLSA<br>SVGDRVTITCRAS<br>QGINNYLSWYQ<br>QKSGKAPKPLIYS<br>ASSLETGVPSRFS<br>GSRSGTDYTLTITS<br>LQPEDIATYYCQQ<br>YNDSPLTFGGGT<br>KVEIKR |
| **WRAIR-5023** | | | |
| CAGGTGCAGCTGCAGGAGTCGGGCCCAGGACTGGTGAAGCCT<br>TCGGAGATCCTGTCCCTCACCTGCGCTGTCTCTGGTGGCTCCAT<br>CAGCGCTGGTTACTACTGGGGCTGGATCCGCCAACACCCAGG<br>GAAGGGGCTGGAGTGGATTGGGAATATCTATGGTAATAGTGC<br>GAACACCTACTACAATCCCTCCCTCAAGAGTCGAGTCACCATTT<br>CAAAAGACACGTCCAAGAACCAGTTCTTCCTGAAGCTGACCTC<br>TGTGACCGCCGCGGACACGGCCGTGTATTACTGTGCGAGAGG<br>GTTCAACTGGTTCGATGTCTGGGGCCCGGGAGTCCTGGTCACC<br>GTCTCCTCA | QVQLQESGPGLVK<br>PSEILSLTCAVSGGSI<br>SAGYYWGWIRQHP<br>GKGLEWIGNIYGNS<br>ANTYYNPSLKSRVTI<br>SKDTSKNQFFLKLTS<br>VTAADTAVYYCAR<br>GFNWFDVWGPGV<br>LVTVSS | GACATTCAGATGACCCAGTCTCCATCCTCCCTGTCTGCTT<br>CTGTAGGAGACAGAGTCACCATCACTTGCCGGGCAAGT<br>CAGGGCATTAGTAGTTATTTAAATTGGTATCAGCAGAA<br>ACCGGGGAAAGCCCCTAAGCTCCTGATCTTTTATGCAAA<br>CCGTTTGGAAAGTGGGGTCCCATCAAGGTTCAGCGGCA<br>GTGGATCTGGGACAGAATTCACTCTCACCATCAGCAGC<br>CTGCAACCTGAGGATTTCGCAACTTATTACTGTCAACAC<br>TATAATAGTTTGCCCATGTACAGTTTTGGCCAGGGGACC<br>AAAGTGGAGATCAAACGG | DIQMTQSPSSLSA<br>SVGDRVTITCRAS<br>QGISSYLNWYQQ<br>KPGKAPKLLIFYA<br>NRLESGVPSRFSG<br>SGSGTEFTLTISSL<br>QPEDFATYYCQH<br>YNSLPMYSFGQG<br>TKVEIKR |
