# Supplementary material for: Diverse array of neutralizing antibodies elicited upon Spike Ferritin Nanoparticle vaccination in rhesus macaques
Source: Nat Commun. 2024 Jan 3;15:200. doi: 10.1038/s41467-023-44265-0 (PMC10764318; doi:10.1038/s41467-023-44265-0)
Supplement: Supplementary file 3 — Reporting Summary [file 41467_2023_44265_MOESM3_ESM.pdf]

nature portfolio

Corresponding author(s): M. Gordon Joyce, Shelly J. Krebs

Last updated by author(s): Nov 30, 2023

Reporting Summary

Nature Portfolio wishes to improve the reproducibility of the work that we publish. This form provides structure for consistency and transparency in reporting. For further information on Nature Portfolio policies, see our Editorial Policies and the Editorial Policy Checklist.

Statistics

For all statistical analyses, confirm that the following items are present in the figure legend, table legend, main text, or Methods section.

n/a	Confirmed	
☐	☒	The exact sample size (*n*) for each experimental group/condition, given as a discrete number and unit of measurement
☐	☒	A statement on whether measurements were taken from distinct samples or whether the same sample was measured repeatedly
☐	☒	The statistical test(s) used AND whether they are one- or two-sided<br>*Only common tests should be described solely by name; describe more complex techniques in the Methods section.*
☒	☐	A description of all covariates tested
☒	☐	A description of any assumptions or corrections, such as tests of normality and adjustment for multiple comparisons
☐	☒	A full description of the statistical parameters including central tendency (e.g. means) or other basic estimates (e.g. regression coefficient) AND variation (e.g. standard deviation) or associated estimates of uncertainty (e.g. confidence intervals)
☐	☒	For null hypothesis testing, the test statistic (e.g. *F*, *t*, *r*) with confidence intervals, effect sizes, degrees of freedom and *P* value noted<br>*Give P values as exact values whenever suitable.*
☒	☐	For Bayesian analysis, information on the choice of priors and Markov chain Monte Carlo settings
☒	☐	For hierarchical and complex designs, identification of the appropriate level for tests and full reporting of outcomes
☒	☐	Estimates of effect sizes (e.g. Cohen's *d*, Pearson's *r*), indicating how they were calculated

Our web collection on statistics for biologists contains articles on many of the points above.

Software and code

Policy information about availability of computer code

Data collection: Diffraction data for the WRAIR-5001-RBD and WRAIR-5021-RBD complexes were collected at Advanced Photon Source (APS), Argonne National Laboratory beamline 24-ID-E and measured using a Dectris Eiger 16M PIXEL detector to a final resolution of 4.3 Å and 2.5 Å, respectively. Diffraction data indexing, integration, and scaling were carried out using the XDS-GUI. Data collection statistics are reported in Table S2.

Data analysis	Neutralization is the geometric mean of the IC50 values calculated using 5-parameter logistic regression from at least two-independent experiments performed in triplicates (R package nplr). Non-parametric Spearman correlations were used to assess relationship between neutralization and binding or neutralization and effector function data as well as between neutralization data obtained from the pseudotyped and authentic SARS-CoV-2 neutralization assays. Survival curves were compared individually to the isotype control antibody using a Mantel-Cox log-rank test. Fold change in binding to mutant proteins was calculated relative to the wild-type WA1/2020 Spike or RBD proteins. In absence of binding, a background binding value (0.05 nm in BLI assays) was attributed. Fold change in neutralization to VOC was calculated relative to the IL1/2020 virus. Non-neutralizing mAbs were assigned the IC50 of 25 µg ml-1 antibody, the mAb starting concentration in the assay. All tests, except for the 5-parameter logistic regression performed in R (version 3.6.3) and R studio (1.2.1355), were performed in Prism (version 9, GraphPad Software). Data were graphed using Prism software (version 9, GraphPad Software).Flow plots were generated using FlowJo version 10. Real-time interactions between purified proteins and antibodies were measured by Biolayer interferometry and analyzed by the FortéBio Data Analysis software 10.0. Structural diffraction data indexing, integration, and scaling were carried out using the HKL2000 suite37. Phenix xtriage (version 1.11.1-2575-0000) was used to analyze all the scaled diffraction data output from HKL2000 (suite 37) and XDS (version Jan 26, 2018 BUILT=20180126), and structure quality was assessed with MolProbity (Phenix suite version 1.11.1-2575-0000). All crystal structures described in this study were solved by molecular replacement using the program Phaser, version 2.139. All structure figures were generated using PyMOL (version 1.3).

For manuscripts utilizing custom algorithms or software that are central to the research but not yet described in published literature, software must be made available to editors and reviewers. We strongly encourage code deposition in a community repository (e.g. GitHub). See the Nature Portfolio guidelines for submitting code & software for further information.

Data

Policy information about availability of data

All manuscripts must include a data availability statement. This statement should provide the following information, where applicable:

- Accession codes, unique identifiers, or web links for publicly available datasets
- A description of any restrictions on data availability
- For clinical datasets or third party data, please ensure that the statement adheres to our policy

All other data are available in the main manuscript, Supplementary Information, or the Source Data file provided with this paper. Items described in this study will be made available to the scientific community by contacting M.G.J. or S.J.K. and upon completion of a materials transfer agreement. The associated accession numbers, coordinates and structure factors of the crystallographic complexes reported in this paper are available from the Protein Data Bank (PDB) with accession codes PDB: 8FI9 (https://doi.org/10.2210/pdb8fi9/pdb) and 8FHY (https://doi.org/10.2210/pdb8fhy/pdb). The antibody sequences are available at Genbank with accession numbers OR078588-OR078627 and are provided in Supplementary Table 6. Antibody variable regions were synthesized and cloned (Genscript) into CMVR expression vectors (NIH AIDS reagent program) between a murine Ig leader (GenBank DQ407610) and the constant regions of human IgG1 (GenBank AAA02914), Igκ (GenBank AKL91145) or Igλ (GenBank AAA02915). Recombinant SARS-CoV-2 proteins RBD (318-514), NTD (1-290) and S1 (1-665) were made from a synthesized full-length Spike sequence (Genscript) from strain USA/IL1/2020 (GenBank # MN988713). Additional sequences used in this work include the coding sequence for SARS-CoV-2 (Genbank # MN908947), Wuhan-Hu-1 strain genome sequence (GenBank # MN9089473) stabilized trimer (S-2P), codon-optimized S expression plasmid encoding SARS-CoV-1 (Sino 1-11, GenBank # AY485277), and IL1/2020 isolate (GenBank # MN988713).

Research involving human participants, their data, or biological material

Policy information about studies with human participants or human data. See also policy information about sex, gender (identity/presentation), and sexual orientation and race, ethnicity and racism.

Reporting on sex and gender	No human data used.
Reporting on race, ethnicity, or other socially relevant groupings	No human data used.
Population characteristics	No human data used.
Recruitment	No human data used.
Ethics oversight	No human data used.

Note that full information on the approval of the study protocol must also be provided in the manuscript.

Field-specific reporting

Please select the one below that is the best fit for your research. If you are not sure, read the appropriate sections before making your selection.

☒ Life sciences ☐ Behavioural & social sciences ☐ Ecological, evolutionary & environmental sciences

For a reference copy of the document with all sections, see nature.com/documents/nr-reporting-summary-flat.pdf

Life sciences study design

All studies must disclose on these points even when the disclosure is negative.

Sample size

Non-human primate (NHP).02 (animal Hs1606375, as previously published ref23) was selected based upon representative binding and neutralization profiles as shown in Supplementary Fig. 1. A single animal was chosen as a minimal sample size to allow identification of antigen-specific antibodies.

Sample size for the K18-hACE2 animal groups was n=13/group, 7 female, 6 male. From this initial group, n=8 animals were assessed for 14 days for weight, clinical signs, and mortality. Calculations indicated that a 60% incidence rate, with a power of 80%, group sizes of 8 animals would be adequate. Post-hoc analysis of the results indicates that this group size was powered for the study.

Data exclusions

No data were excluded from the analysis.

Replication

Binding and neutralization data were calculated from two independent experiments, performed in triplicate. Neutralization potencies obtained from each assay correlated for all mAbs tested, and all findings were replicated. For the prophylactic protection studies, on day −1, groups of 13 K18-hACE2 mice (8-10 weeks of age) were injected intravenously with the purified antibodies at the indicated dose of 10 mg ml-1. On study day 0, all mice were inoculated with 1.25×104 PFU of SARS-CoV-2 Delta B.1.617.2 via intranasal instillation, a challenge dose determined from a previous study (ref 81). All mice were monitored from study day 0 to study day 1, with body weight measurements taken every day, twice daily, every 12 h, out to day 10. At 48 hours post-challenge, 5 K18-hACE2 animals from each group were sacrificed for viral replication analysis.
Antibody affinity measurements were carried out in duplicate, with at least four concentrations used for each of the RBD molecules. ELISA assays were carried out in duplicate for each assay, with at least one replicate experiment also completed.
All attempts at replication were successful.

Randomization

Mice were allocated to the groups randomly. The selection of the rhesus macaque was based upon high neutralization compared to other animals.

Blinding

Rhesus macaque Hs1606375 was chosen for monoclonal antibody isolation based upon high neutralization compared to other animals using identical criteria. The Principal Investigators were blinded to the treatment and sham groups for the in vivo mouse passive protection studies until the studies were complete and results were available. Apart from the mouse experiments, there were no other group allocations, and as such the Principal Investigators were not blinded.

Reporting for specific materials, systems and methods

We require information from authors about some types of materials, experimental systems and methods used in many studies. Here, indicate whether each material, system or method listed is relevant to your study. If you are not sure if a list item applies to your research, read the appropriate section before selecting a response.

Materials & experimental systems

n/a	Involved in the study
☐	☒ Antibodies
☐	☒ Eukaryotic cell lines
☒	☐ Palaeontology and archaeology
☐	☒ Animals and other organisms
☒	☐ Clinical data
☒	☐ Dual use research of concern
☒	☐ Plants

Methods

n/a	Involved in the study
☒	☐ ChIP-seq
☐	☒ Flow cytometry
☒	☐ MRI-based neuroimaging

Antibodies

Antibodies used

The MM43 monoclonal antibody (SinoBiological, #40591-MM43) was used for primary staining of Spike ferritin nanoparticle (SpFN) antigen-specific B cells. For Flow cytometry: Mouse anti-human IgG, HRPconjugated (Southern Biotech, Catalog 9040-05, Lot J3314.T085 and L0717-ZC27B, Clone JDC-10, Dilution 1/2800), Goat antimouse IgG, HRP conjugated (Southern Biotech, Catalog 1030-05, Lot K3515-T566E, Dilution 1/6000), Mouse anti-human CD3, BV510 conjugated (BD Biosciences Cat# 563918, Lot 7037566, Clone SP34-2, Dilution 1/20), Mouse anti-human CD4, BV510 conjugated (BD Biosciences Cat# 562970, Lot 7094727, Clone SK3, Dilution 1/80), Mouse anti-human CD8, BV510 conjugated (BioLegend Cat# 301047, Lot B221676, Clone RPA-T8, Dilution 1/80), Mouse anti-human CD14, BV510 conjugated (BioLegend Cat# 301841, Lot B236875, Clone M5E2, Dilution 1/80), Mouse anti-human CD16, BV510 conjugated (BD Biosciences Cat#563830, Lot 7103547, Clone 3G8, Dilution 1/160), Mouse anti-human CD56, BV510 conjugated (BioLegend Cat# 318339, Lot B205718, Clone HCD56, Dilution 1/40), Mouse anti-human CD19, ECD conjugated (Beckman Coulter Cat# IM2708U, Lot 97, Clone J3-119, Dilution 1/40), Mouse anti-human IgG, BV785 conjugated (BD Biosciences Cat# 564230, Lot 7037913, Clone G18-145, Dilution 1/80), Mouse anti-human IgD, APC-Cy7 conjugated (BioLegend Cat# 348217, Lot B234185, Clone IA6-2, Dilution 1/160), Mouse anti-human IgM, PE-Cy5 conjugated (BD Biosciences Cat# 551079, Lot 7041529, Clone G20-127, Dilution 1/10).

Validation	All antibodies from commercial sources undergo validation using flow cytometry, western blot, chromatin immunoprecipitation, immunofluorescence, immunohistochemistry, and/or biofunctional assays to ensure specificity and to provide clarity for research uses. Specifically, mouse anti-human CD3, BV510 conjugated (Clone SP34-2), mouse anti-human CD16, BV510 conjugated (Clone 3G8), mouse anti-human IgG, BV785 conjugated (Clone G18-145), mouse anti-human IgM, PE-Cy5 conjugated (Clone G20-127) and mouse anti-human CD4, BV510 conjugated (Clone SK3) underwent stringent testing and validation by BD Biosciences to assure that it generates a high-quality conjugate with consistent performance and specific binding activity. The validation process included testing on a combination of primary cells, cell lines and/or transfectant cell models with relevant controls using multiple immunoassays to ensure biological accuracy. BD also performs multiplexing with additional antibodies to interrogate antibody staining in multiple cell populations. Mouse anti-human CD8, BV510 conjugated (Clone RPA-T8), mouse anti-human CD14, BV510 conjugated (Clone M5E2), mouse anti-human CD56, BV510 conjugated (Clone HCD56) and mouse antihuman IgD, APC-Cy7 conjugated (Clone IA6-2) were thoroughly validated by Biolegend. Each antibody was tested using the following criteria: 1.Staining of 1-3 target cell types with either single- or multi-color analysis detailed in the QC specification (including positive and negative controls). The tested cells can be primary cells and/or cell lines known to be positive or negative for the target antigen; 2.Each batch product is validated by QC testing with a series of dilutions to make sure the product is working within expected antibody titer range; 3.Each batch is compared to an internally established "gold standard" to maintain batch-to-batch consistency; 4. When applicable, our products are side-by-side tested with our competitors' products to make sure that BioLegend's products exceed or are at least the same quality; 5.For most tandem dye-conjugated products, color compensation is examined in order to verify tandem integrity.<br>General statements from Biolegend:<br>"The specificity and sensitivity of each antibody is thoroughly validated in the New Product Development stage. This is done by staining multiple target cells with either single- or multi-color analysis or by other testing approaches. The QC specifications and testing SOPs and gold standard for each product are then developed".<br>In general, each product is tested using the following criteria:<br>1. Staining of 1-3 target cell types with either single- or multi-color analysis detailed in the QC specification (including positive and negative controls). The tested cells can be primary cells and/or cell lines known to be positive or negative for the target antigen.<br>2. Each batch product is validated by QC testing with a series of dilutions to make sure the product is working within expected antibody titer range.<br>3. Each batch is compared to an internally established "gold standard" to maintain batch-to-batch consistency.

Eukaryotic cell lines

Policy information about cell lines and Sex and Gender in Research

Cell line source(s)	HEK293T/17 (ATCC #CRL-11268) (Human female origin), HEK293-ACE2 (Integral Molecular) (Human female origin), Vero E6 (ATCC #CRL-1586) (female Chlorocebus sabaeus origin (African green monkey)), Expi293F (ThermoFisher Scientific #A14527) (Human female origin), Freestyle 293F (ThermoFisher Scientific #R79007) (Human female origin), THP-1 (Millipore #88081201) (human male origin) cell lines were utilized in this study.
Authentication	All cell lines were authenticated using short-tandem repeat analysis.
Mycoplasma contamination	Cell lines were not recently tested for mycoplasma contamination.
Commonly misidentified lines (See ICLAC register)	No commonly misidentified cell lines were used in this study.

Animals and other research organisms

Policy information about studies involving animals; ARRIVE guidelines recommended for reporting animal research, and Sex and Gender in Research

Laboratory animals	Transgenic mice, B6.Cg-Tg(K18-ACE2)2Prlmn/J, common name: K18-hACE2, male, female, 8-10 weeks of age. Sample size for the K18-hACE2 animal groups was n=13/group, 7 female, 6 male. This group size was sufficient to provide clear differences between viral and animal clinical outcomes. Animals were maintained in IVC cages on negatively pressurized Allentown PNC racks that were HEPA filtered and directly vented though the building's exhaust system. The racks and animal rooms were negatively pressurized. Access to all facilities is controlled electronically by the buildings management systems and restricted to approved users only. The environment, temperature, and humidity, within the animal facility room were constantly monitored by the building management system. Temperatures were also monitored and recorded daily in individual animal rooms by animal care staff using electronic thermometers. All temperature set points were within The Guide recommended range of 68-79°F for mice. Acceptable Institutional daily fluctuations are between 67-74°F with a humidity range of 30-70%. Light cycles in all animal holding and procedure spaces are controlled on a 12/12 light/dark cycle.<br><br>PBMCs from Non-human primate (NHP).02 (animal Hs1606375, as previously published ref23).
Wild animals	This study did not involve wild animals.
Reporting on sex	Mice were randomized evenly per group between male and female mice, with a total of 13 mice per group (7 female, 6 male). Non-human primate (NHP).02 (animal Hs1606375, as previously published ref23) was a 4-year-old male.<br><br>Research-naive Chinese-origin rhesus macaques (age 3 to 7 years) were distributed—on the basis of age, weight, and sex—into four cohorts of eight animals in study ref23. In the animals immunized with SpFN used for binding studies were four females and four Indian origin rhesus macaques (age 3 to 15 years) were distributed—on the basis of age, weight, and sex as described in study ref38.

Field-collected samples | This study did not involve samples collected from the field.

Ethics oversight | Research was conducted in compliance with the Animal Welfare Act and other federal statutes and regulations relating to animals and experiments involving animals and adheres to principles stated in the Guide for the Care and Use of Laboratory Animals, NRC Publication, 1996 edition. Material has been reviewed by the Walter Reed Army Institute of Research. The opinions or assertions contained herein are the private views of the authors, and are not to be construed as official, or as reflecting true views of the Department of the Army or the Department of Defense. The research protocol was approved by the Institutional Animal Care and Use Committee of the Trudeau Institute, protocol 20-007, and USAMRDC Animal Care and Use Review Office (ACURO). K18-hACE2 transgenic mice were obtained from Jackson Laboratories (Bar Harbor, ME). Mice were housed in the animal facility of the Trudeau Institute and cared for in accordance with local, state, federal, and institutional policies in a National Institutes of Health American Association for Accreditation of Laboratory Animal Care-accredited facility.

Note that full information on the approval of the study protocol must also be provided in the manuscript.

Plants

Seed stocks | N/A

Novel plant genotypes | N/A

Authentication | N/A

Flow Cytometry

Plots

Confirm that:

☒ The axis labels state the marker and fluorochrome used (e.g. CD4-FITC).

☒ The axis scales are clearly visible. Include numbers along axes only for bottom left plot of group (a 'group' is an analysis of identical markers).

☒ All plots are contour plots with outliers or pseudocolor plots.

☒ A numerical value for number of cells or percentage (with statistics) is provided.

Methodology

Sample preparation | Cryopreserved PBMCs from naïve or SpFN vaccinated rhesus macaques were thawed in warm media containing benzonase, then washed with PBS and stained for viability using the Aqua Live/Dead stain (ThermoFisher) and Fc receptors blocked using normal mouse IgG (Invitrogen #02-6502). After centrifuging, cells were then stained for 30 min at RT for SARS-CoV-2 and SARS-CoV-1 reactive B cells by incubating with SpFN and SpFN1 particles, followed by a wash with staining buffer (1x PBS containing with FCS and NaN3). Cells were then incubated at RT for 30 min with fluorochrome conjugated secondary antibodies specific to the S protein of either SARS-CoV-2 (WRAIR-2054-APC) or SARS-CoV-1 (mm02-PE, SinoBiological #40150-MM02). Finally, after another wash, cells were incubated at RT for 30min with a cocktail of phenotyping antibodies including CD3 BV510 (BD Biosciences Cat# 740187, Lot# 0261796), CD4 BV510 (BD Biosciences Cat# 562970, Lot# 0177290), CD14 BV510 (BioLegend Cat# 301842, Lot# B264335), and CD16 BV510 (Biosciences Cat# 563830, Lot# 1307468) as dump channel markers, and CD19 PE-Cy5 (Beckman Coulter Cat# IM2643U Lot# 200100), CD20 AlexaFluor700 (BD Biosciences Cat# 560631 Cat# 134775), IgG PE-Cy7 (BioLegend Cat# 410721 Lot# M1310G05), IgM BV650 (Biolegned Cat# 314525 Lot# B294373). SARS-CoV-2 RBD, and NTD (ThermoFisher) that had been biotinylated, tetramerized and conjugated to streptavidin-PE were also included in this step. Viable, dump channel -, IgG+, IgM-, CD19+ B cells that were antigen positive to either a SpFN particle or tetramerized subdomain, or a combination, were single-cell sorted into PCR plates containing lysis buffer composed of murine RNAse inhibitor (New England Biolabs), dithiothreitol (DTT), SuperScript III First Strand Buffer (ThermoFisher), Igepal (Sigma), and carrier RNA (Qiagen) at one cell per well using a FACS ARIA (Becton Dickinson) and stored at -80°C until subsequent reverse transcription. Analysis was performed using FlowJo 10 (BD Bioscience).

Instrument | FACSAria (Becton Dickinson)

Software | FlowJo, version 10

Cell population abundance | Less than 5% of the total B cell population was specific for SARS-CoV-2 antigens or spike ferritin nanoparticles (SpFN). All antigen- positive B cells were sorted into lysis buffer, sequenced, cloned and characterized.

Gating strategy | CD19+/IgG+/IgD-/IgM- B cells reactive to SARS-CoV-2 antigens, or SpFN particles were sorted directly into lysis buffer at one cell per well into PCR plates using a FACSAria (Becton Dickinson) and stored at −80°C until subsequent reverse transcription. Increased frequencies of antigen-specific and cross-reactive B cells were detected in Donor #3, following SARS-CoV-2

infection compared to pre-pandemic PBMC samples. Gating strategy was supplied in Extended Data Fig. 1.

☒ Tick this box to confirm that a figure exemplifying the gating strategy is provided in the Supplementary Information.
